# Foraging and metabolic consequences of semi-anadromy for an endangered estuarine fish

**DOI:** 10.1371/journal.pone.0173497

**Published:** 2017-03-14

**Authors:** Bruce G. Hammock, Steven B. Slater, Randall D. Baxter, Nann A. Fangue, Dennis Cocherell, April Hennessy, Tomofumi Kurobe, Christopher Y. Tai, Swee J. Teh

**Affiliations:** 1 Aquatic Health Program, School of Veterinary Medicine, Department of Anatomy, Physiology, and Cell Biology, VetMed 3B, University of California Davis, Davis, California, United States of America; 2 California Department of Fish and Wildlife, Stockton, California, United States of America; 3 Department of Wildlife, Fish and Conservation Biology, University of California Davis, Davis, California, United States of America; Uppsala Universitet, SWEDEN

## Abstract

Diadromy affords fish access to productive ecosystems, increasing growth and ultimately fitness, but it is unclear whether these advantages persist for species migrating within highly altered habitat. Here, we compared the foraging success of wild Delta Smelt—an endangered, zooplanktivorous, annual, semi-anadromous fish that is endemic to the highly altered San Francisco Estuary (SFE)—collected from freshwater (<0.55 psu) and brackish habitat (≥0.55 psu). Stomach fullness, averaged across three generations of wild Delta Smelt sampled from juvenile through adult life stages (n = 1,318), was 1.5-fold higher in brackish than in freshwater habitat. However, salinity and season interacted, with higher fullness (1.7-fold) in freshwater than in brackish habitat in summer, but far higher fullness in brackish than freshwater habitat during fall/winter and winter/spring (1.8 and 2.0-fold, respectively). To examine potential causes of this interaction we compared mesozooplankton abundance, collected concurrently with the Delta Smelt, in freshwater and brackish habitat during summer and fall/winter, and the metabolic rate of sub-adult Delta Smelt acclimated to salinities of 0.4, 2.0, and 12.0 psu in a laboratory experiment. A seasonal peak in mesozooplankton density coincided with the summer peak in Delta Smelt foraging success in freshwater, and a pronounced decline in freshwater mesozooplankton abundance in the fall coincided with declining stomach fullness, which persisted for the remainder of the year (fall, winter and spring). In brackish habitat, greater foraging ‘efficiency’ (prey items in stomachs/mesozooplankton abundance) led to more prey items per fish and generally higher stomach fullness (i.e., a higher proportion of mesozooplankton detected in concurrent trawls were eaten by fish in brackish habitat). Delta Smelt exhibited no difference in metabolic rate across the three salinities, indicating that metabolic responses to salinity are unlikely to have caused the stomach fullness results. Adult migration and freshwater spawning therefore places young fish in a position to exploit higher densities of prey in freshwater in the late spring/summer, and subsequent movement downstream provides older fish more accessible prey in brackish habitat. Thus, despite endemism to a highly-altered estuary, semi-anadromy provided substantial foraging benefits to Delta Smelt, consistent with other temperate migratory fish.

## Introduction

Animal movement is a major driver of species abundance and distributions, making its causes and consequences important to ecology, evolutionary biology and conservation [1]. Migrations are large-scale movements of animals during which individuals are more likely to ignore stimuli from resources than during non-migratory movement [2]. Migrations have evolved as mechanisms to exploit heterogeneous and predictable distributions of conditions, resources, mates, and natural enemies across landscapes, thereby increasing fitness [3, 4]. For example, wolves migrate seasonally to follow caribou herds [5], elk migrate to track high quality forage [6], and pregnant bighorn sheep migrate to areas with poor quality forage to avoid predation on their offspring [7].

Migration of diadromous fishes involves movement between fresh and saltwater, and is thought to have evolved to increase survival of larvae and juveniles and to improve the foraging success of adults [8, 9]. Diadromous species include anadromous fishes, which spend most of their lives in saltwater and return to freshwater to spawn, and catadromous fishes which exhibit the opposite life history strategy [8]. Gross et al. [10] provides compelling evidence for the bottom-up portion of the hypothesis as outlined by Moyle and Cech [8]: the authors found that catadromy is a far more common life history strategy at low latitudes where oceans are less productive than freshwater, whereas anadromy is more common at higher latitudes where marine ecosystems are more productive. Kedney et al. [11] found support for the top-down portion of the hypothesis [8], observing that Threespine Stickleback eggs and males in brackish habitat were subject to higher predation rates than their freshwater counterparts.

Here we focus on the bottom-up portion of the migration evolution hypothesis [8]. Foraging success is essential because food intake is an important driver of growth, with faster growth rates associated with increased fitness due both to increased survival and fecundity ([10] and references therein). Researchers commonly compare migratory to non-migratory individuals of partially anadromous or partially semi-anadromous species (i.e., fishes in which only a portion of the population migrates to marine or brackish habitats) to better understand the causes and consequences of migration. There are numerous examples from mid and higher latitude regions of improved foraging success, increased size, and increased fecundity of migratory fish compared to non-migratory fish. For example, as productivity in freshwater increases the probability of anadromy decreases in partially migratory Arctic Char [12]. Wysujack et al. [13] described similar results from a mesocosm experiment in Sweden in which food limitation increased the proportion of migratory Brown Trout. Improved foraging of migratory fishes scales up to improve fitness correlates, likely offsetting the risks associated with migration [1]. For example, several studies have demonstrated increased fecundity for anadromous temperate salmonids (e.g., [14–16]), and in southeastern Alaska, anadromous Dolly Varden were larger and far more fecund than freshwater residents [17]. The pattern of improved fitness correlates of fish migrating to saline habitat appears to hold for semi-anadromous species as well (i.e., fish that migrate to brackish rather than marine habitat). For example, Kerr et al. [18] observed that semi-anadromous White Perch grew larger than freshwater residents in Chesapeake Bay. While temperate anguillid eels are catadromous and are thus an exception to the biogeographic pattern described by Gross et al. [10], the eels nevertheless grew faster in both brackish and marine environments than in freshwater [19]. Thus, at temperate latitudes and above, ecological theory predicts that migratory fish will exhibit a foraging advantage over freshwater residents. However, whether this foraging advantage persists for temperate fish migrating to highly altered saline habitat is unclear.

We addressed this question in Delta Smelt (*Hypomesus transpacificus*), a small, zooplanktivorous, annual fish that is ideal for several reasons. First, the species is semi-anadromous, moving from freshwater to brackish habitat in the late spring as larvae and juveniles and back to freshwater in the winter/spring as adults prior to the spawning period [20, 21]. The landward migration coincides with increased turbidity during winter, potentially allowing Delta Smelt to avoid predation while migrating [22]. Second, the species is both partially migratory (only a subset of the population migrates) and semi-anadromous [21]. This allowed us to compare the foraging success of fish in freshwater and brackish habitat throughout much of their life-cycle (juvenile-adult) and thus make inferences regarding the foraging consequences of migration [23]. Third, the species is endemic to the San Francisco Estuary (SFE), which has several long-term monitoring datasets, including mesozooplankton [24], allowing us to examine long-term trends in Delta Smelt prey abundance in both freshwater and brackish habitats. Finally, the Delta Smelt is protected under the federal and state Endangered Species Acts but its small range overlaps with the most important water-supply hub in California, making the species tremendously important to a variety of agencies and stake-holders [25–27]. Finally, the SFE is highly altered and studied, with large, well-documented changes to its hydrology, physical habitat, and biota (e.g., [28–30]).

Of the many changes to the SFE, the 1986 introduction of the invasive clam *Potamocorbula amurensis* may exert the strongest influence on the foraging consequences of migration for Delta Smelt [31]. Chlorophyll a declined five-fold in brackish portions of the SFE following the introduction of *P*. *amurensis*, moving secondary productivity from the water column (i.e., zooplankton) to the benthos [28, 29]. In possible consequence, juvenile Delta Smelt in Suisun Bay, the most saline portion of its recent juvenile range, exhibited relatively poor nutritional status and condition [32]. If nutritional stress is widespread and persists throughout the year in brackish regions, the habitat type may represent an ecological trap for Delta Smelt [33]. That is, Delta Smelt may have evolved migratory behavior to exploit abundant food resources in brackish regions, but long-term declines in those resources have turned a beneficial life-history strategy into a maladaptive one. Specifically, we hypothesized that severe declines in brackish zooplankton densities have caused Delta Smelt in brackish habitat to have lower foraging success than freshwater Delta Smelt. Alternatively, despite its endemism to a highly altered estuary [30, 34], semi-anadromy may still afford the foraging benefits expected for a temperate fish [10, 18, 19].

We assessed these hypotheses by comparing mesozooplankton abundance and foraging success of wild Delta Smelt in freshwater and brackish regions across seasons. The influence of season was explicitly examined because seasonal changes in food availability and predation pressure are strong drivers of migration for many animals, including fishes (e.g., [5, 35]). Then, we explored several possible drivers of foraging success of Delta Smelt, including prey weight, prey count, and a measure of foraging efficiency (i.e., the ratio of prey items in Delta Smelt stomachs to mesozooplankton abundance in the water column) in freshwater and brackish habitat across seasons. We also examined the correlation between stomach fullness and both salinity and mesozooplankton density in the water column (i.e., functional response; [36]). However, differences in metabolic demand due to salinity could affect stomach fullness via food conversion efficiency [37] and gastric evacuation rate [38], potentially influencing the apparent foraging consequences of migration. Therefore, we also performed a laboratory experiment to determine whether the metabolic rate of Delta Smelt is influenced by salinities that overlap the natural range of Delta Smelt (i.e., 0.4, 2.0, and 12.0 psu).

## Materials and methods

### Ethics statement

A California Endangered Species Act (CESA) Memorandum of Understanding (MOU) was made and entered into by and between Swee Teh of the University of California, Davis (permittee) and the California Department of Fish and Wildlife (CDFW). The purpose of this CESA MOU was to authorize the permittee to obtain and possess Delta Smelt (*Hypomesus transpacificus*) collected by the CDFW Interagency Ecological Program for scientific purposes pursuant to Fish and Game Code (FGC) 2081 (a). The portions of the study relating to Delta Smelt were approved by the Institutional Animal Care and Use Committee, University of California, Davis and followed the methodology in the approved protocol (IACUC Protocol #18175).

### Long-term mesozooplankton abundance

We used the California Department of Fish and Wildlife (CDFW) long-term monitoring Zooplankton Study dataset from 1972–2015 to characterize how mesozooplankton, a major food resource of Delta Smelt, varies between freshwater and brackish regions both on a decadal and seasonal time scale [39]. During the ongoing survey, mesozooplankton were collected during 10 min bottom-to-surface oblique tows using a mesozooplankton net (160 μm mesh size) at sites in the upper SFE [24]. Following collection, mesozooplankton were preserved in formalin and taken to a CDFW laboratory in Stockton CA to be identified and enumerated. Detailed field and laboratory methods, including a site map, are available online (http://www.water.ca.gov/bdma/meta/zooplankton.cfm). Some changes to the sampling design have occurred during the 44 years of monitoring, so to make the dataset temporally and spatially comparable we excluded some sampling events and sites from our analysis. Specifically, data from the second mesozooplankton sample of each month were excluded where applicable since mesozooplankton sampling was performed twice per month until 1993 and once per month thereafter. Sampling of mesozooplankton in the winter (Dec, Jan and Feb) was conducted inconsistently, so data from these surveys were also excluded, as were sites that were not sampled consistently through time beginning in 1972. These exclusions left 12 sites that were sampled monthly from Mar-Nov (Table 1), including 2 station pairings where stations sampled from 1972 through 1993 were replaced by nearby stations sampled from 1994 through 2015 (station 80 was replaced by station D16 2.8 km away, and station 42 was replaced by station D6 2.2 km away; Table 1). CDFW started counting cumaceans and ostracods in 2001 and 2002, so both taxa were excluded from the mesozooplankton analysis. To examine decadal trends in mesozooplankton abundance in freshwater and brackish habitat, sites were divided between freshwater (<0.55 psu) and brackish (≥0.55 psu) habitat based on mean salinities for each site, and annual mean mesozooplankton abundance (mesozooplankton m^-3^) was calculated for both salinity categories and plotted by year. 0.55 psu represents the lower salinity boundary of the Low Salinity Zone, which is the approximate boundary of landward salinity intrusion in estuaries [40, 41]. To show how mesozooplankton abundance in the SFE varies seasonally, monthly means of mesozooplankton abundance were plotted in four categories, freshwater (<0.55 psu) and brackish (≥0.55 psu) from 1972–1986 (before the invasion of *P*. *amurensis*; [28]) and freshwater and brackish from 1987–2015 (after the invasion). By summing across mesozooplankton taxa our aim was to prevent taxonomic complexity from obscuring large changes in mesozooplankton abundance. We acknowledge that mesozooplankton m^-3^ is an imperfect proxy for food availability for Delta Smelt, as Delta Smelt can prey upon organisms that are not sampled effectively by the mesozooplankton net (e.g., larval fish, mysids, amphipods; S2–S4 Figs), exhibit positive selection for certain organisms (i.e., *Eurytemora affinis* and *Pseudodiaptomus forbesi*; [39]), and rarely (<1% of juvenile-adult Delta Smelt in this study) prey upon some of the organisms sampled by the mesozooplankton survey (rotifers, crab zoea, and barnacle nauplii). To address the latter point we made a second plot (S1 Fig) that excluded rotifers, crab zoea, and barnacle nauplii from the mesozooplankton, leaving only taxa commonly found in Delta Smelt stomachs (copepods and cladocera; S2–S4 Figs). For more taxonomically detailed analyses of decadal trends in zooplankton abundance and community structure see [42] and [24].

**Table 1:** Station IDs, the number of monthly CDFW mesozooplankton samples, mean salinity (psu) and mean temperature (°C) for each site (SD in parentheses) sampled Mar-Nov from 1972–2015. The first 8 sites are considered brackish (≥0.55 psu) in our analyses and final 4 are freshwater (<0.55 psu).

Station ID	n	Salinity (SD)	Temp (SD)
28	381	6.43 (4.5)	17.92 (3.0)
32	382	4.84 (3.7)	18.63 (3.4)
42/D06	382	10.53 (5.7)	17.61 (2.8)
48	377	5.03 (3.9)	17.96 (3.0)
54	378	3.47 (3.1)	18.03 (3.1)
60	380	1.97 (2.1)	18.33 (3.1)
64	379	0.70 (1.0)	17.99 (3.3)
74	364	1.26 (1.4)	18.40 (3.2)
80/D16	387	0.30 (0.3)	18.44 (3.4)
86	382	0.11 (0.1)	18.66 (3.6)
92	378	0.25 (0.1)	20.42 (4.3)
D28	372	0.19 (0.1)	19.37 (3.9)

### Delta Smelt foraging

CDFW began routine monitoring of pelagic fish abundance in the SFE in 1959 with the Summer Townet Survey (STN; [43]). The STN sampled 40 stations per survey every other week from Jun-Aug during the present study (mesh size: 0.25 cm mesh at the cod end). The Fall Midwater Trawl (FMWT) was initiated in 1967 [44, 45] and sampled 122 stations during monthly surveys from Sep-Dec throughout the present study (mesh size: ranges from 20.3 cm stretch mesh at the mouth to 1.3 cm at the cod end). The Spring Kodiak Trawl (SKT) began in 2002 and sampled 40 stations per month from Jan-May during the present study [21] (mesh size: ranges from 5.1 cm stretch mesh at the mouth to 0.6 cm at the cod end). During STN and FMWT (but not SKT), a mesozooplankton sample was collected concurrently with the fish trawls at a subset of 32 stations using the same mesozooplankton net as the Zooplankton Study (160 μm mesh size). CDFW surveys measured specific conductance (μS/cm), temperature (°C), and turbidity (Nephelometric Turbidity Units; NTU) of surface water at each station, and specific conductance data were converted to salinity. Mesozooplankton were preserved and processed in the same manner as in the Zooplankton Study described above. Detailed survey methods, including site maps, are described on CDFW’s website (https://www.wildlife.ca.gov/Regions/3) and by Honey et al. [46]. As above, the abundances of each individual taxa were summed to provide an indicator of food availability for Delta Smelt (mesozooplankton m^-3^). Trawl samples were collected at sites over navigable inland waters of CA from San Pablo Bay upstream through the SFE. It was unnecessary to obtain permission to access sites because the locations are public waterways. Take of Delta Smelt by CDFW was permitted via the Interagency Ecological Program under the Section 7 Biological Opinion issued to the U.S. Bureau of Reclamation by the U.S. Fish and Wildlife Service in 1996, and additional amendments directly from the U.S. Fish and Wildlife Service to CDFW.

In summer 2011 UC Davis staff began accompanying CDFW and flash-freezing Delta Smelt that were caught during STN, FMWT, and SKT in liquid nitrogen and transporting them to UC Davis’ Aquatic Health Laboratory (Davis, CA). Over the nearly three-year project, Delta Smelt used in this study were collected from 55 sites between Aug 2011 and May 2014 (S1 Table). At UC Davis, an array of endpoints that reflect fish health and condition were measured on the three year-classes [47]. In this study, we used body weight, stomach fullness, mean prey item mass, and prey item counts from the fish, and associated mesozooplankton abundance and water quality measurements collected during the surveys (see [32] for details). During laboratory necropsies, individuals were weighed and fork length was measured as each fish thawed. Mean fork lengths of fish used in this study in freshwater and brackish habitats were 40.1 and 41.9 mm during STN, 60.2 and 57.6 mm during FMWT, and 67.5 and 66.0 mm during SKT, respectively. We note that while flash-freezing decreases the fork length and weight of Delta Smelt, the effect is small (<1%; [47]). Fish were rapidly dissected (~5–10 min per fish), and the gastro-intestinal tract was preserved in 70% ethanol and sent to CDFW’s Diet Study Laboratory for analysis (Stockton, CA). At CDFW, stomach contents were weighed, identified, and enumerated, with lengths recorded for larger prey items (i.e., amphipods, mysids and fish). Wet weight of prey in stomachs was determined either by multiplying the count of each prey type by a wet weight estimate, or from lengths using length-weight equations for larger zooplankton. Weights of the various prey types for each stomach were summed, and stomach fullness was calculated as the weight of the stomach contents divided by fish body weight multiplied by 100 [39]. The average mass per prey item for each fish was calculated by dividing the weight of the gut contents by the number of prey items. A measure of foraging ‘efficiency’ was calculated as the number of prey items in the stomach divided by mesozooplankton density (mesozooplankton m^-3^) in concurrent zooplankton tows. We used prey ‘count’ rather than prey ‘biomass’ in this calculation because differences in stomach fullness between freshwater and brackish habitat were driven mainly by the number of prey in stomachs rather than biomass (Results). Finally, for fish with non-zero stomach fullness, the percentage of the total stomach content weight was calculated for each taxon for freshwater and brackish habitat for STN, FMWT, and SKT, plotted, and included in the Supporting Information (i.e., diet analysis; n = 1,264; S2–S4 Figs).

### Metabolic demand

Chabot et al. [48] defines standard metabolism as the minimal amount of oxygen needed by a fish to support its aerobic metabolic rate. The salinity at which standard metabolism is minimized may reflect a physiological optimum, as well as the salinity at which food conversion efficiency peaks (e.g., [37, 49]). The Delta Smelt used in our experiment were cultured at 0.4 psu at the Fish Conservation and Culture Laboratory (Byron, CA), which uses a genetic management strategy to maintain genetic similarity to the wild population and prevent inbreeding [50], and transported to UC Davis where the experiment was conducted. Once at UC Davis, fish were acclimated to three salinity treatments (0.4 psu [n = 18], 2.0 psu [n = 20] and 12.0 psu [n = 24]) for a minimum of three weeks. This period was chosen to provide sufficient time for Delta Smelt to acclimate to the higher salinities [51]. For reference, 0.1 psu was the minimum salinity at which Delta Smelt were collected during CDFW trawls between summer 2011 and 2014 (n = 1,933). In the same dataset, 99.7% of Delta Smelt were caught at <12 psu, with a maximum of 15.6 psu (although wild Delta Smelt have been recorded up to 18 psu; [52]). 2 psu is a mid-range salinity that is of significance to resource managers (i.e., X2; [53, 54]) and to which Delta Smelt exhibit lower levels of stress detected via gene expression analysis [55]. 0.4 psu was the salinity of the well water in the lab where the experiment was performed, and the salinity at which the fish were raised. Mean salinity, temperature, specific conductivity and pH of the treatment tanks are in Table 2.

**Table 2:** Temperature (°C), salinity (psu), specific conductivity (μS/cm) and pH of the treatment tanks averaged across the acclimation and experimental period for the metabolism experiment. Standard deviations are in parentheses.

Treatment	Salinity (SD)	Temp (SD)	Sp. Cond (SD)	pH (SD)
0.4 psu	0.4 (0.00)	16.12 (0.48)	870.8 (16.3)	8.37 (0.19)
2.0 psu	2.01 (0.03)	16.11 (0.10)	3,821 (39)	8.62 (0.05)
12.0 psu	12.01 (0.06)	15.88 (0.07)	20,060 (100)	8.52 (0.07)

Metabolic measurements were made using intermittent respirometry in 1.1 L acrylic plastic chambers [48, 56]. Measurements were collected from individual fish placed into one of four respirometers immersed in an aerated, 300 L, insulated water bath completely surrounded by black plastic sheeting. Each evening we randomly selected and set the water bath to one of the salinities to which the fish were acclimated (0.4, 2.0, or 12 psu). Water was kept at 15.9°C (SD = 0.1) during the experiment, the same temperature to which the fish were acclimated (Table 2). Blanks were run during the night and metabolism measurements began the following morning, using fish that were acclimated to the salinity in the water bath. The fish were given 40 min to acclimate to the respirometers before the first of five 30 min measurement cycles began. Dissolved oxygen measurements were made using galvanic oxygen probes, and measurements were recorded using Loligo Systems AutoResp software. The body weight of fish used in metabolic measurements ranged from 0.94 to 3.33 g. Standard metabolic rate was calculated from the slope of the relationship between dissolved oxygen and time, minus the slope of the blank (see S1 File for detailed methods).

### Statistical analysis

We tested whether mesozooplankton abundance declined through time using linear regression. Mesozooplankton count data were log_10_-transformed to linearize the relationship between mesozooplankton count and year. The transformed count data were the response variable and ‘year’ was the predictor variable. Data from freshwater and brackish sites were analyzed separately to determine whether the slopes differed (Table 1).

We used a 2×3 ANOVA to examine how stomach fullness varied seasonally in freshwater and brackish regions. The response variable was proportion stomach fullness that had been arcsine-square root transformed to improve normality. The interaction was between salinity (freshwater [<0.55 psu] and brackish [≥0.55 psu]) and survey (Summer Townet [STN], Fall Midwater Trawl [FMWT], and Spring Kodiak Trawl [SKT]). The three surveys were used as predictors to both account for systematic differences in sampling among the surveys and to allow us to examine seasonal effects (STN: summer, FMWT: fall/winter, and SKT: winter/spring). For both this and the foraging analyses of wild fish below we used the salinity at which each Delta Smelt was caught to assign it to a salinity bin (freshwater or brackish). Because stomach fullness increased rapidly early in the morning and gradually thereafter, time of day was included as a discrete variable with five levels following Hammock et al. [32]. The levels were 6–8:00, 8:00–10:00, 10:00–12:00, 12:00–14:00, and 14:00–16:00. Year-class was also included as a discrete variable to account for any year-to-year differences in foraging success [32]. Because we were specifically interested in the difference in stomach fullness between freshwater and brackish habitat, planned linear contrasts between the salinity habitats for each of the three surveys were performed. Sample size was 1,318, with 148, 132, and 402 fish in STN, FMWT, and SKT in freshwater, and 122, 167, and 347 fish in STN, FMWT, and SKT in brackish habitat.

To determine whether stomach fullness was driven by number of prey, weight of prey, or both, we used an ANCOVA. The response variable was proportion stomach fullness that had been arcsine-square root transformed, and the predictors were year-class, number of prey/fish, time of day, and mean weight of a prey items/fish (n = 1,281).

Next, we performed four analyses with similar predictors but different response variables to identify the drivers of the stomach fullness factorial results, all of which included a salinity by survey interaction (2×3 interaction). Significant interactions for all four analyses were followed by planned linear contrasts to separate means of interest. In the first, mesozooplankton abundance (collected concurrently to the fish tows; mesozooplankton m^-3^) that was log_10_-transformed to reduce heterogeneity of variance was the response variable in an ANOVA, and year-class, survey, salinity (fresh/brackish), and a survey by salinity interaction were the predictors. For STN, n = 28 tows in freshwater and 35 tows in brackish habitat, and for FMWT n = 17 and 20 tows for the freshwater and brackish habitat (note: mesozooplankton tows did not accompany fish collection during SKT). In the second analysis, we used the same set of predictors in an ANOVA to examine mean prey item weight per fish. For STN, n = 143 and 105 fish, for FMWT n = 131 and 164 fish, and for SKT n = 396 and 342 fish in freshwater and brackish habitats, respectively. In the third analysis, prey item count per fish was the response variable in an ANOVA, and year-class, salinity (fresh/brackish), time of day, survey, and the interaction between survey and salinity were the predictors. For STN, n = 148 and 122 fish; for FMWT, n = 132 and 167 fish; and for SKT, n = 402 and 347 fish in freshwater and brackish habitats, respectively. In the fourth analysis, prey items/mesozooplankton m^-3^ was the response variable in an ANOVA, and year-class, survey, salinity (fresh/brackish), and a survey by salinity interaction were the predictors. For STN, n = 148 and 102 fish for freshwater and brackish habitats, and for FMWT, n = 70 and 72 fish for freshwater and brackish habitats.

We performed a second analysis on the stomach fullness data in which Delta Smelt (n = 1,318) were divided among six salinity bins rather than two, allowing us to examine the influence of salinity (or its correlates) on foraging success at finer resolution. The salinity bins were <0.55, 0.55–2, 2–4, 4–6, 6–8, and >8 psu. We ran an ANOVA in which proportion stomach fullness (arcsine-square root transformed) was the response variable, and year-class, time of day, and salinity bin were the independent variables. We used a Tukey HSD mean comparison to separate the salinity bin means. Sample sizes for each salinity bin from lowest to highest salinity were 682, 193, 186, 112, 105, and 40.

To determine whether prey density correlated with stomach fullness (i.e., functional response), we first excluded the 97 fish caught between 6:00 and 8:00 from the 1,318 fish for which we had stomach fullness data because stomach fullness was likely to be more strongly influenced by nighttime fasting than prey density soon after sunrise. We also excluded 847 fish with stomach fullness data for which a mesozooplankton tow did not accompany the fish tow (mainly from SKT), and 5 Delta Smelt which had larval fish in their stomachs (because we lacked abundance data on larval fish), leaving 369 Delta Smelt. We then averaged stomach fullness for all fish associated with each mesozooplankton tow, since individual fish could not be considered independent replicates. The dataset was then split between freshwater (<0.55 psu) and brackish (≥0.55 psu) habitat and the stomach fullness measurements were binned by mesozooplankton abundance: low, medium and high abundance bins in both freshwater and in brackish habitats. The two habitat types were analyzed separately because zooplankton communities differ substantially between freshwater and brackish habitat [24], and Delta Smelt were more efficient predators in brackish habitat (see Results). The bins were defined by the rank of mesozooplankton abundance for each tow, 15 tows per bin in freshwater, and 15–16 tows per bin in brackish habitats. There were 58, 76 and 83 fish in the low, medium and high mesozooplankton abundance freshwater bins and 54, 48 and 50 fish in the brackish mesozooplankton abundance bins. We used two one-way ANCOVAs to determine whether stomach fullness varied with mesozooplankton bin, one for freshwater and one for brackish habitat. As above, an arcsine-square root transformation was applied to proportion stomach fullness and used as the response variable. In each ANCOVA, mesozooplankton bin and year-class were discrete predictor variables, and time of day was the covariable. Tukey HSD mean separations of the mesozooplankton bins followed a significant ANCOVA.

Means of temperatures and turbidities are reported for freshwater and brackish habitats for each survey, weighted by the number of fish. The means include all sampling events during which we collected Delta Smelt for which we measured stomach fullness (n = 1,318).

We analyzed the metabolic data in two steps. To determine whether the 40 min acclimation period provided sufficient time for mg O_2_ h^-1^ (standard metabolic rate) to stabilize for individual fish, we compared oxygen consumption (mg O_2_ h^-1^) among the five sequential measurement cycles using an ANCOVA. Fish wet weight was the covariable following Chabot et al.[48], and measurement cycle (1–5) was the discrete independent variable. Weight was significant (ANCOVA, F_1, 300_ = 75.7451, P <0.0001), but measurement cycle was not (F_4, 300_ = 1.8219, P = 0.1245), suggesting that standard metabolic rate did not vary systematically among the measurement cycles. However, though statistically insignificant, mean standard metabolic rate did decline numerically until the final two measurement periods (means for the five periods were: 1.01, 0.98, 0.95, 0.93, 0.93 mg O_2_ h^-1^). Therefore, to further ensure that our results were not altered by a possible influence of acclimation period, we ran three ANCOVAs in which the response variable (mg O_2_ h^-1^) was calculated based on a mean of all five measurement cycles (presented in the main text because ‘measurement cycle’ was insignificant), the final four measurement cycles (following [48]), and only the final two measurement cycles, the periods during which metabolic rate was numerically minimized. The latter two ANCOVA results are presented in S2 File. In all three ANCOVAs, standard metabolic rate (mg O_2_ h^-1^) was the response variable, body weight was the covariable, and salinity was the discrete independent variable. In addition, we tested whether respirometry chamber and ‘experiment day’ (a proxy for age of the fish) were useful predictors, but neither was significant so they were removed from the final analysis. Treatment means are presented after adjusting for the effect of mass using the body weight parameter estimate and mean body weight for each treatment. In addition, unadjusted treatment means are presented in S2 File. An additional ANOVA was used to determine whether there were significant treatment differences in body weights of fish measured following the respirometry (n = 62; salinity was the only predictor). All statistical analyses were performed using JMP Pro 12.0.01 software.

## Results

**Fig 1:**
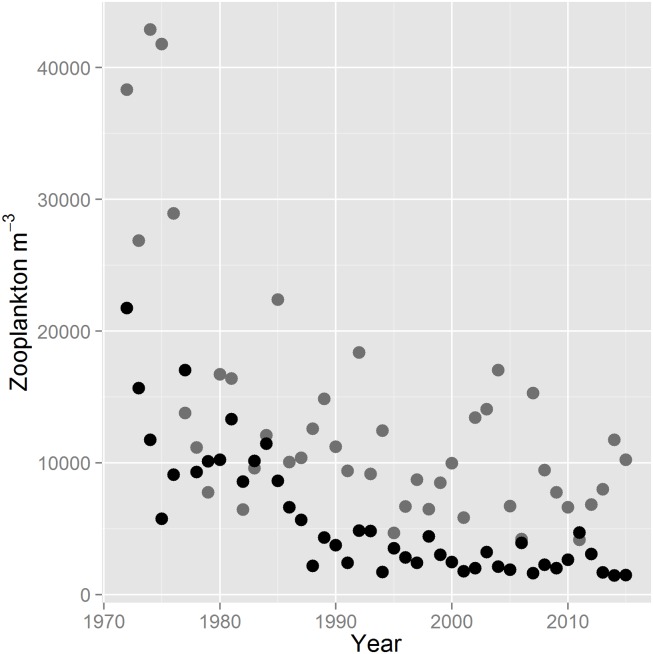
Mean mesozooplankton m^-3^ by year (1972–2015) in the SFE. Grey points represent freshwater (<0.55 psu mean salinity) and black points represent brackish habitat (≥0.55 psu mean salinity).

Based on CDFW’s long-term mesozooplankton monitoring data, mesozooplankton abundance has declined since the 1970s in both freshwater (linear regression, F_1, 42_ = 24.3400, P < 0.0001) and brackish habitat in the SFE (linear regression, F_1, 42_ = 109.9700, P < 0.0001). The steepest decline occurred in brackish regions, as the slope parameter estimate was -0.0116 (95% CI: -0.0162, -0.070) in freshwater and -0.0219 (95%CI: -0.026, -0.0178) in brackish habitat. Copepods and cladocerans (a subset of the mesozooplankton) also declined in both freshwater (linear regression, F_1, 42_ = 9.869, P = 0.003077) and brackish habitat (linear regression, F_1, 42_ = 70.72, P < 0.0001; S1 Fig). Based on three-year averages of the mesozooplankton data, abundance declined from 36,027 mesozooplankton m^-3^ from 1972–1974 to 9984 from 2013–2015 in freshwater, or 3.6-fold. In brackish habitat, mean mesozooplankton abundance declined from 16,394 to 1535 mesozooplankton m^-3^ over the same period, or 10.7-fold. Averaged over 2011–2014, roughly the period of our wild Delta Smelt dataset, mesozooplankton abundance was 2.8-fold higher in freshwater habitat of the SFE (although this comparison excludes Dec-Feb). Averaged across the 44 year dataset, copepods and cladocerans (major Delta Smelt prey items; [39],S2–S4 Figs) comprised 45.5 and 36.1% of the freshwater mesozooplankton, respectively; and 73.2 and 3.8% of the brackish mesozooplankton, respectively. Crab zoea, rotifers, and barnacle nauplii (non-prey taxa, S2–S4 Figs) comprised 18.3 and 23.1% of the mesozooplankton in freshwater and brackish habitats, respectively (Note: Fig 1 includes these taxa and S1 Fig does not). Mesozooplankton were also more abundant in freshwater in the mesozooplankton tows that occurred concurrently with the Delta Smelt tows, with 6,025 mesozooplankton m^-3^ in freshwater and 2,727 in brackish (2.2-fold).

While mesozooplankton were generally more abundant in freshwater, when averaged across the entire stomach fullness dataset (n = 1,318), stomach fullness was 1.54-fold higher for Delta Smelt caught in brackish habitat than in freshwater (ANOVA, F_1, 1306_ = 9.8223, P = 0.0018). Stomach fullness also changed significantly with time of day (ANOVA, F_4, 1306_ = 22.3024, P<0.0001), increasing during the day (0.13 from 6–8:00, 0.39 from 8:00–10:00, 0.44 from 10:00–12:00, 0.55 from 12:00–14:00, and 0.57 from 14:00–16:00). However, the difference in stomach fullness between freshwater and brackish habitat was not due to systematic differences in the time of collection. The average sampling times in freshwater and brackish habitats were similar (10:34 and 10:53, respectively), and of the 1,318 fish for which we collected stomach fullness data, 6.2 and 8.6% were caught before 8:00 in freshwater and brackish regions, respectively (when stomach fullness is substantially lower). The improvement in foraging success exhibited by fish in brackish regions was consistent over the three year study, as stomach fullness was 1.64, 1.33 and 1.41-fold higher in brackish regions for the 2011, 2012 and 2013 year-classes.

**Fig 2:**
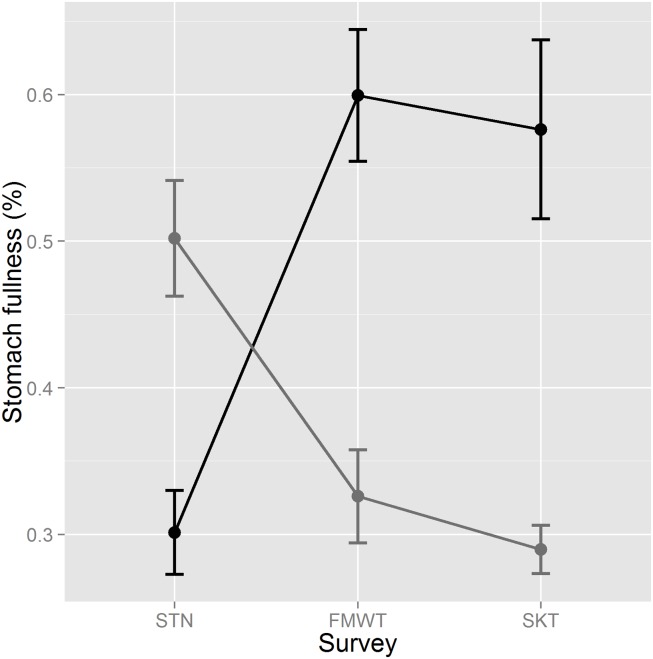
Mean stomach fullness (%) for Delta Smelt by survey and salinity. STN = Summer Townet, FMWT = Fall Midwater Trawl, SKT = Spring Kodiak Trawl. Grey represents stomach fullness of Delta Smelt sampled from freshwater (<0.55 psu) and black represents stomach fullness of Delta Smelt sampled from brackish habitat (≥0.55 psu). Mean differences between freshwater and brackish habitat were significant for each survey. Error bars are ±SE, n = 1,318.

Although mean stomach fullness was substantially higher in brackish regions, this difference was not consistent seasonally, as survey (STN, FMWT, and SKT) and salinity (freshwater vs brackish) interacted significantly (ANOVA, F_2,1306_ = 21.9754, P <0.0001; Fig 2). Stomach fullness was 1.67-fold higher in freshwater during the STN survey (i.e., summer; linear contrast, P = 0.0005), but 1.84 and 1.99-fold higher in brackish water in the FMWT (fall/winter) and SKT (winter/spring) surveys (P <0.0001 for both linear contrasts; Fig 2). Stomach fullness also varied by year-class (ANOVA, F_2,1306_ = 5.7962, P = 0.0031), with fish in the 2013 year-class having significantly higher stomach fullness than the previous two year-classes (mean stomach fullness was 0.40, 0.39, and 0.55 for 2011, 2012 and 2013 year-classes, respectively; Tukey HSD).

In STN (summer), the dominant prey items for Delta Smelt in both freshwater and brackish habitats were copepods, comprising 92.3 and 79.1% of prey weight in stomachs, with *Pseudodiaptomus* contributing the greatest mass (S2 Fig). Copepods remained the dominant prey items by weight in FMWT (fall/winter), comprising 65.7% of prey weight in FMWT in freshwater and 60.2% in brackish, although the dominant genera diverged: *Pseudodiaptomus* was dominant in freshwater and *Acartiella sinensis* in brackish water (S3 Fig). In Spring Kodiak Trawl (winter/spring), copepods remained the dominant prey item in freshwater, comprising 59.9% of the stomach contents by weight. However, in brackish habitats the two prey items that together comprised the greatest proportion of stomach content weight were the crustaceans Cladocera (22.5%) and cumaceans (18.9%), and copepods declined to 38.5% of stomach content weight (S4 Fig).

Stomach fullness was influenced by year-class (ANCOVA, F_2, 1272_ = 5.9054, P < 0.0028), increased with time of day (ANCOVA, F_4, 1272_ = 17.0252, P < 0.0001), and increased with prey item count (F_1, 1272_ = 563.4816, P < 0.0001) and mean weight of prey items (ANCOVA, F_1, 1272_ = 282.6974, P < 0.0001). Thus, both number and size of prey were important drivers of foraging success.

**Fig 3:**
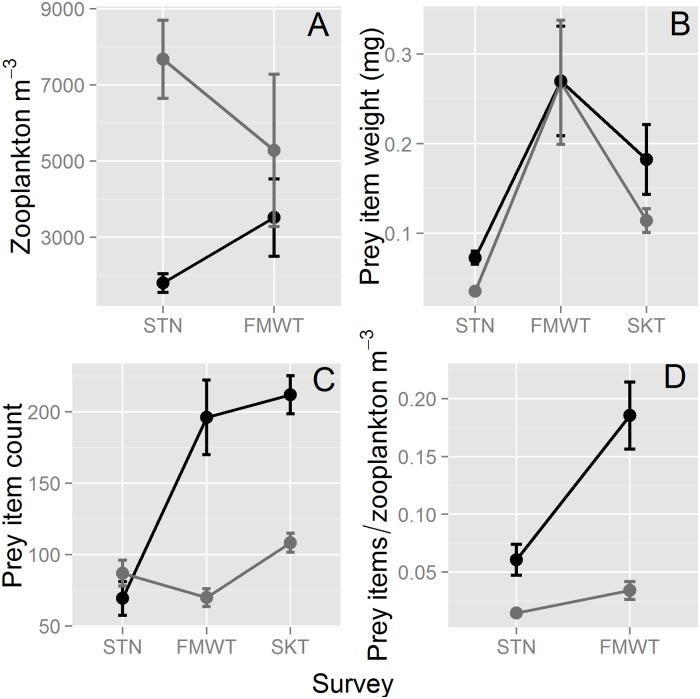
Mesozooplankton abundance and Delta Smelt foraging. Grey is freshwater (mean salinity <0.55 psu), black is brackish habitat (≥0.55 psu), and error bars are ±SE. (A) mesozooplankton abundance by survey (collected concurrently with Delta Smelt), (B) mean weight (mg) of prey items in Delta Smelt stomachs by survey, (C) mean number of prey items by survey in Delta Smelt stomachs, and (D) the number of prey items in Delta Smelt stomachs per mesozooplankton m^-3^ by survey. STN is the Summer Townet survey (summer), FMWT is the Fall Midwater Trawl survey (fall/winter), and SKT is the Spring Kodiak Trawl survey (winter/spring).

Mesozooplankton abundance (collected concurrently with Delta Smelt during STN and FMWT surveys) did not vary with year-class (ANOVA, F_2, 94_ = 0.5867, P = 0.5582), did not vary by survey (ANOVA, F_1, 94_ = 1.6481, P = 0.2024), but did vary by salinity, with higher abundances in freshwater than brackish habitat overall (ANOVA, F_1, 94_ = 19.7221, P <0.0001), and survey and salinity interacted (ANOVA, F_1, 94_ = 11.9940, P = 0.0008; Fig 3A). Mesozooplankton abundance was higher in freshwater during summer (linear contrast, P <0.0001), and there was no significant difference between freshwater and brackish habitat in the fall/winter (linear contrasts, P = 0.4923; Fig 3A). Mesozooplankton density was significantly lower in fall/winter than in summer in freshwater (linear contrast, P = 0.0022) but the corresponding increase in mean abundance from summer to fall in brackish habitat was not significant (linear contrast, P = 0.1923). In the long-term mesozooplankton monitoring dataset, mesozooplankton declined slightly from summer into fall in brackish habitat (S5 Fig). In contrast, the seasonal decline of freshwater mesozooplankton in tows associated with Delta Smelt was not anomalous, as it has occurred (on average) throughout freshwater portions of the SFE since the 1970s (S5 Fig). Thus, there is strong evidence of declining mesozooplankton abundance from summer into fall/winter in freshwater, and either no change or a slight increase in brackish habitat.

The mean weight of prey items in Delta Smelt stomachs varied by year-class (ANOVA, F_2, 1273_ = 14.3173, P < 0.0001) and by survey (ANOVA, F_2,1273_ = 15.4494, P < 0.0001), but not by salinity (ANOVA, F_1,1273_ = 1.2595, P = 0.2620), and survey and salinity did not interact (ANOVA, F_2, 1273_ = 0.9561, P = 0.3847; Fig 3B). Mean prey item weight was significantly higher than the other two surveys during FMWT, and was significantly lower than the other surveys during STN (Tukey HSD). Mean prey item weight was significantly higher for the 2012 year class (0.30 mg item^-1^) than the 2011 and 2013 year classes (0.09 and 0.17 mg item^-1^; Tukey HSD). Although the difference was not statistically significant, mean weight of prey items was higher in brackish habitat (0.13 mg item^-1^ in freshwater and 0.19 mg item^-1^ in brackish).

The number of prey items in Delta Smelt stomachs varied by year-class (ANOVA, F_2, 1306_ = 11.5540, P<0.0001), was higher in brackish habitat (ANOVA, F_1,1306_ = 38.7752, P <0.0001), varied by survey (ANOVA, F_2, 1306_ = 17.1472, P <0.0001), increased with time of day (ANOVA, F_4, 1306_ = 46.4058, P < 0.0001), and survey and salinity interacted (ANOVA, F_2, 1306_ = 19.1597, P < 0.0001; Fig 3C). There was no difference in prey per fish during summer between freshwater and brackish habitats (linear contrast, P = 0.8227), but the prey item count was significantly higher in brackish habitat in both fall/winter (2.8-fold, linear contrast, P <0.0001, FMWT) and winter/spring (2.0-fold, linear contrast, P = 0.0029, SKT; Fig 3C). Overall, the number of prey items per fish was 1.87-fold higher in brackish regions, was highest in the 2013 year-class (Tukey HSD), and was highest in SKT (Tukey HSD).

Prey items/mesozooplankton m^-3^ (‘foraging efficiency’) did not vary by year-class (ANCOVA, F_2, 382_ = 0.6196, P = 0.5387), but increased during the day (ANCOVA, F_1,382_ = 9.5478, P < 0.0001), was significantly higher in brackish habitat (ANCOVA, F_1, 382_ = 60.9706, P <0.0001), increased significantly from STN to FMWT (ANCOVA, F_1, 382_ = 4.6053, P = 0.0325), and survey and salinity interacted (ANCOVA, F_1, 382_ = 12.7813, P = 0.0004; Fig 3D). Prey items/mesozooplankton m^-3^ was significantly higher in brackish habitat in both summer (4.2-fold, linear contrast, P = 0.0013) and in fall/winter (5.5-fold, linear contrast, P <0.0001; Fig 3D).

**Fig 4:**
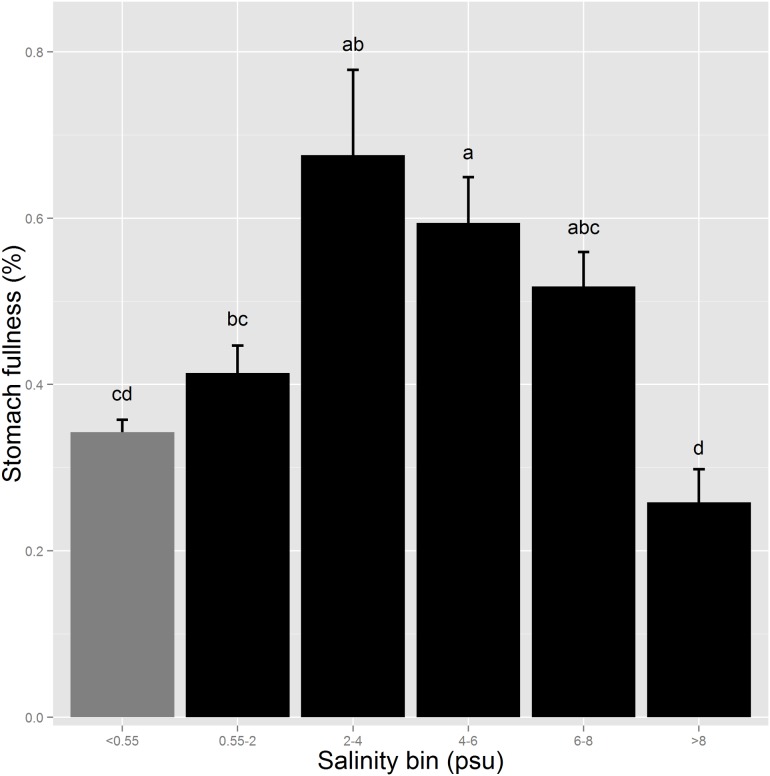
Mean stomach fullness (%) of Delta Smelt by salinity bin. The grey bar represents freshwater (<0.55 psu) and the black bars represent brackish habitat (≥0.55 psu). Sample sizes for each salinity bin from lowest to highest salinity = 682, 193, 186, 112, 105, and 40. Differing letters denote statistically significant differences. Error bars are SEs.

For the analysis in which the stomach fullness data was divided among 6 salinity bins (<0.55, 0.55–2, 2–4, 4–6, 6–8, and >8 psu) rather than 2 (<0.55 and ≥0.55 psu), stomach fullness peaked at 2–4 psu (Fig 4). Stomach fullness varied with year-class (ANOVA, F_2, 1306_ = 4.4561, P = 0.0118), time of day (ANOVA, F_4, 1306_ = 29.4295, P <0.0001), and salinity bin (ANOVA, F_5, 1306_ = 11.1834, P <0.0001). Stomach fullness was significantly reduced at both low (<0.55 psu) and at high (>8 psu) salinities (Fig 4). Relatively few fish fell in the highest salinity bin (40 of 1,318), explaining why mean stomach fullness was relatively high in brackish habitat in the analyses above (≥0.55 psu). Mean turbidity in the six salinity bins was 31.8, 53.2, 39.1, 28.9, 28.0, and 52.9 NTU.

**Fig 5:**
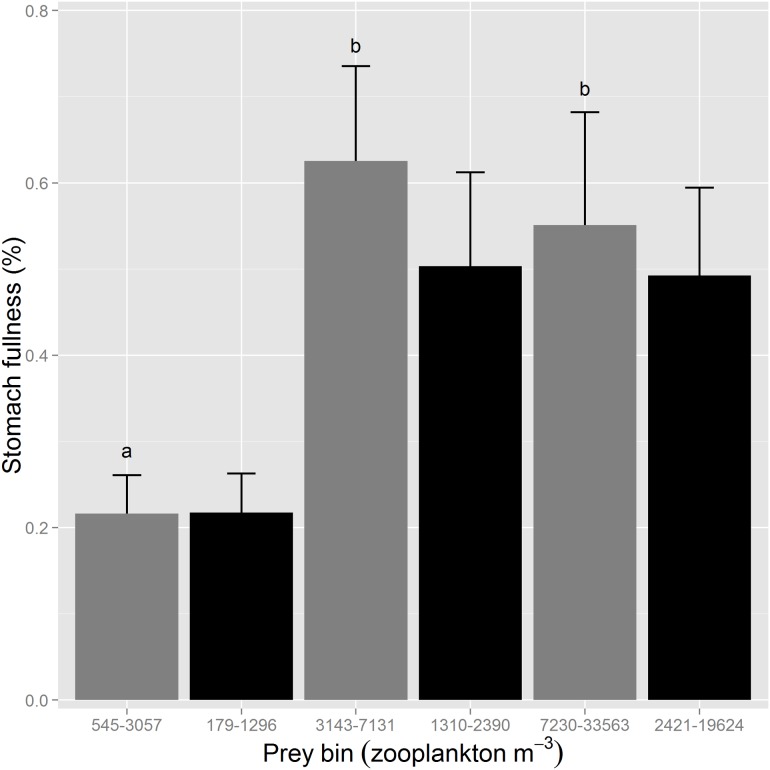
Mean stomach fullness for wild Delta Smelt by mesozooplankton density. Grey bars represent freshwater (<0.55 psu) and black bars represent brackish habitat (≥0.55 psu). Bins of low, medium, and high mesozooplankton abundance were defined by the rank of mesozooplankton abundance of each tow, 15 tows per bin in freshwater, and 15–16 tows per bin in brackish habitat. The number of fish that fell in each bin, in order of increasing mesozooplankton abundance, is 58, 76 and 83 in freshwater and 54, 48 and 50 in brackish. Note that we only tested for differences in means within freshwater or brackish habitat and that the x-axis is not continuous or to scale. Differing letters indicate statistically significant differences (there was no significant difference between the three brackish habitat means). Error bars are SEs.

The relationship between stomach fullness and prey density was consistent with a type II (satiating) functional response ([36]; Fig 5). In freshwater, stomach fullness varied significantly and positively with mesozooplankton bin (i.e., density; ANCOVA, F_2, 39_ = 5.7275, P = 0.0066) but not by time of day (ANCOVA, F_1, 39_ = 0.9799, P = 0.3283) or year-class (ANCOVA, F_2, 39_ = 0.3699, P = 0.6932). In brackish water, stomach fullness did not vary significantly with mesozooplankton bin (ANCOVA, F_2, 41_ = 2.3879, P = 0.1045) or with time of day (ANCOVA, F_1, 41_ = 0.1656, P = 0.6861), and year-class was significant (ANCOVA, F_2, 41_ = 3.6878, P = 0.0337). Stomach fullness was highest for the 2011 year-class and lowest for the 2013 year-class (Tukey HSD).

Weighted mean water temperature was 14.4°C (n = 682) in freshwater and 13.4°C (n = 636) in brackish water. Weighted mean water temperatures for STN, FMWT, and SKT, averaged across years, were 21.4, 13.4 and 12.2°C in freshwater and 20.4, 14.2, and 10.5°C in the brackish water stations, respectively. Weighted mean water temperatures for the 2011, 2012, and 2013 year-classes were 12.8, 14.8, and 15.7°C. Weighted mean turbidities for freshwater and brackish habitats were 31.8 and 40.6 NTU, respectively. Weighted mean turbidities for STN, FMWT, and SKT, averaged across years, were 33.8, 30.8, and 31.5 NTU and 51.7, 31.3 and 41.2 NTU for freshwater and brackish water, respectively.

Following metabolic rate determinations, fish generally appeared healthy and robust upon their removal from the respirometers, and no mortality occurred during the measurements. Based on means of all five measurement cycles for each fish, body wet weight influenced standard metabolic rate (ANCOVA, F_1, 58_ = 19.1853, P <0.0001) and salinity did not (ANCOVA, F_2, 58_ = 1.1322, P = 0.3294). Treatment means, adjusted by mean weight of each treatment using the parameter estimate for weight in the ANCOVA, were 0.489, 0.555, and 0.477 mg O_2_ h^-1^. Excluding neither the first nor the first three measurement cycles altered the results (S2 File). No difference was detected in fish weight between the three salinity treatments (ANOVA, F_2, 59_ = 0.2167, P = 0.8058). Mean standard metabolic rate in the experiment was 0.506 mg O_2_ h^-1^ (SD = 0.20) and mean body wet weight was 1.97 g (SD = 0.53).

## Discussion

The SFE is among the most altered estuaries in the world [30, 34]. Among other changes, zooplankton abundance has declined substantially, its community composition has changed considerably since the 1970s, and recent work shows that the abundances of several taxa peak earlier in the year (Fig 1, [24, 42, 57]). In terms of food resources for zooplanktivorous fish like the Delta Smelt, the decline is likely more significant than Fig 1 indicates because mean mesozooplankton size has also declined [24], reducing foraging success of SFE fish [31]. Given that the decline in mesozooplankton abundance is most pronounced in brackish regions (Fig 1), and because of recent evidence for nutritional stress in the most saline portion of the summer range of Delta Smelt [32], we hypothesized that semi-anadromy does not confer the foraging benefits predicted by theory [10] for Delta Smelt. Instead, we found that fish in brackish regions had substantially higher foraging success, as stomach fullness was 1.5-fold higher in brackish than freshwater habitats averaged across all seasons. Juveniles did appear to be under nutritional stress in parts of their brackish habitat in summer (Fig 2), consistent with Hammock et al. [32], but for most of the year, foraging success was higher in brackish regions. Below we discuss possible causes for this pattern, but what is immediately evident from our results is that access to freshwater habitat is important to the foraging success of juvenile Delta Smelt, while brackish habitat (2–8 psu) is important for the foraging success of juvenile, sub-adult and adult fish, and migration provides individuals with access to both.

Gross et al. [10] observed that mid and high latitude fishes tend to be anadromous, likely because marine ecosystems are more productive than freshwater at these latitudes, while the opposite is true at low latitudes. The foraging improvement exhibited by Delta Smelt in brackish habitat is therefore consistent with other migratory temperate fishes (e.g., [12, 19]). This suggests that the biogeographic pattern described by Gross et al. [10] is remarkably robust, applying also to an endangered species endemic to a highly altered estuary. The only apparent inconsistency with Gross et al. [10] for Delta Smelt is that foraging success was actually higher in freshwater in the summer. One possibility is that Delta Smelt spawn in freshwater to provide young better access to higher densities of mesozooplankton in the spring and summer (Fig 3A and S5 Fig), and migrate to brackish regions to gain access to potentially larger (Fig 3B), and more available prey most of the year (Fig 3D). Therefore, assuming no costs of migration (e.g., mortality, loss of foraging time), it appeared to be a beneficial life history strategy over our three year study. However, improved foraging does not preclude the possibility of other benefits of migration, such as improved survival of larval fish in freshwater (e.g., lower predation rates) [8]. Whatever the drivers of migration of Delta Smelt to freshwater to spawn, our results indicate that an improvement in foraging success in brackish habitat, due mainly to greater prey capture efficiency (Fig 3D), is likely an important driver of Delta Smelt migration to brackish habitat.

The metabolic results indicate that the differences in stomach fullness between freshwater and brackish habitats were unlikely to have been caused by differences in osmoregulatory costs. We found no evidence that salinity influenced the metabolic demand of Delta Smelt following acclimation, as there was no difference in standard metabolic rate among the 0.4, 2.0 and 12.0 psu treatments. Such a result may not be surprising given recent physiological work in Delta Smelt showing that while gene expression was altered following transfer from 2.3 to 18 psu, there was no corresponding reduction in condition factor following a two week exposure [58]. Delta Smelt are also quite tolerant of high salinity, surviving at 34 psu for at least two weeks [59], although with reduced condition factor [58]. There is also increasing evidence that osmoregulation makes up a relatively small proportion of the energy budget of most fishes (roughly 10%; [60]). For example, the metabolic costs of NaCl transport for Cutthroat Trout is <4% in both fresh and salt water [61]. Thus, the causes for the variation in stomach fullness with salinity do not appear to be related to differential costs of osmoregulation.

Seasonal changes in prey size cannot explain why foraging success was higher in freshwater in the summer but far higher in brackish water in fall, winter and spring (i.e., the crossover interaction in Fig 2). Both fresh and brackish water fish were foraging on prey of nearly identical average weights in the FMWT (fall/winter; Fig 3B), and the overall difference in mean prey weight between freshwater and brackish habitat was not significant. Instead, it was likely caused by an increase in the consumption rate of prey in brackish habitat, as fish in brackish water had approximately double the number of prey items in their stomachs as fish in freshwater much of the year (Fig 3C). This is somewhat paradoxical because mesozooplankton densities were similar in the two habitat types during fall/early winter in tows taken concurrently to the Delta Smelt sampling (Fig 3A), and lower throughout the estuary in brackish habitat during the fall (S5 Fig). Thus, Delta Smelt were more ‘efficient’ predators in brackish water regions (Fig 3D), consuming a substantially higher proportion of available prey. This foraging ‘efficiency’ is the most likely explanation for why brackish fish exhibit generally greater stomach fullness despite generally lower mesozooplankton densities in brackish habitat.

Understanding why Delta Smelt are more efficient predators in brackish regions is an important question, but it is difficult to address based on our results. Brackish water fish exhibited major seasonal shifts in diet, progressing from *Pseudodiaptomus* in summer to *Acartiella sinensis*, mysids, and *Pseudodiaptomus* in the fall/winter to cladocera and cumaceans in the winter/spring (S2–S4 Figs). In freshwater, Delta Smelt remained relatively dependent on the copepods *Pseudodiaptomus* and *Sinocalanus doerrii* year-round (S2–S4 Figs). Thus, one possibility is that while mesozooplankton abundance is generally lower in brackish habitat, brackish regions may have a higher abundance of certain, preferred prey types, or prey that are more easily captured. The prey selectivity analysis used by Slater and Baxter [39] could be applied to Delta Smelt and mesozooplankton collected from freshwater and brackish habitat to examine this hypothesis. Another possibility is that the Delta Smelt are less stressed at salinities between 2–8 psu, making them more efficient predators at mid-range salinities (Fig 4). Hasenbein et al. [55] reported that the transcription of genetic markers for glutathione-s-transferase (an indicator of oxidative stress), heat shock protein 70kDa (general stress), and pro-opiomelanocortin (cortisol production) were minimized following 2 h exposures to 2 and 6 psu compared to 0 (control), 12 and 15 psu. This is consistent with Swanson et al. [62], which references higher survival of Delta Smelt during transport at 8 psu than in freshwater. Thus, it is possible that lower stress levels in Delta Smelt exposed to mid-range salinities (2–8 psu) is contributing to the improved foraging efficiency we observed in brackish regions.

Water temperature and turbidity are two other key abiotic variables that could have influenced stomach fullness and foraging efficiency. As an ectotherm, the metabolism of Delta Smelt increases with temperature until a critical thermal limit is reached [63], increasing gastric evacuation rate (e.g., [38, 64]). The critical thermal maximum for Delta Smelt juveniles ranges from ~27–29°C, depending on the acclimation temperature [59]. Therefore, metabolic demand due to temperature was likely slightly higher in freshwater than in brackish habitat in the summer, as weighted mean temperature during tows in which Delta Smelt were caught was one degree higher in freshwater (21.4 vs 20.4°C). Nevertheless, stomach fullness was higher in the summer in freshwater. In fall, brackish habitats were warmer than freshwater and stomach fullness was significantly higher, while in winter/spring water temperature was warmer in freshwater but stomach fullness remained higher in the brackish regions (Fig 2). Thus, temperature was poorly correlated with stomach fullness and cannot easily explain the large seasonal shifts in stomach fullness between freshwater and brackish habitats. Overall, gastric evacuation rate was likely somewhat higher in freshwater because mean temperature was 1°C higher in freshwater. For reference, Vinagre et al. [38] found that a 1°C increase in water temperature increased the gastric evacuation rate of juvenile *Solea senegalensis* by ~12% between 14 and 20°C. In terms of turbidity, Delta Smelt exhibit an optimal feeding rate between 25 and 80 NTU [65] and mean turbidity in both freshwater and brackish habitats both fell within this range (31.8 and 40.6 NTU in fresh and brackish water). Similarly, Baskerville-Bridges et al. [66] found that Delta Smelt were ineffective predators in clear water. Thus, it is possible that the somewhat higher turbidities in brackish habitat, particularly in the 0.55–4 psu range at which both turbidity and stomach fullness peaked, improved the foraging efficiency of Delta Smelt. Increased turbidity could increase efficiency by improve the visual acuity of Delta Smelt, or perhaps cuing their prey to move up into the water column [67].

The best correlate we found for Delta Smelt stomach fullness in freshwater and brackish habitats was mesozooplankton density, with stomach fullness increasing with increasing density before reaching an asymptote, a type II functional response (Fig 5; [36]). Type II functional responses are difficult to observe in the wild because consumers avoid patches with low food densities, and if prey is consistently abundant in patches with predators, the predators will be satiated [36]. Nevertheless, stomach fullness increased significantly with mesozooplankton density in freshwater, indicating that the low fullness measurements from the fall winter and spring in freshwater habitats were due, at least in part, to low prey abundance (Figs 3C and 5). However, we do not suggest that a threshold density of mesozooplankton at which Delta Smelt will be satiated can be identified based on Fig 5, either in freshwater or brackish habitat. Mesozooplankton abundance was somewhat conflated with maturity (i.e., it was the sub-adult and adult fish in freshwater that exhibited poor foraging success and occupied habitat with relatively low mesozooplankton densities), so we cannot discern to what extent poor foraging success resulted from low prey abundance or small prey size, since larger predacious fishes depend on larger prey [68, 69]. Instead, what Figs 2, 3C and 5 suggest is that higher densities of mesozooplankton would improve foraging success of freshwater Delta Smelt, particularly in fall, winter and spring. A similar, although statistically insignificant, pattern existed in brackish regions (Fig 5), and there is evidence of nutritional stress in the most saline portions of Delta Smelt habitat in the summer [32]. Thus, foraging success may also be limited by low mesozooplankton abundance in brackish regions, but is likely less pronounced than in freshwater in fall, winter and spring. It may also be more regionally specific [32], given the smaller effect size and lack of statistical significance of the functional response in brackish habitat.

Figs 2 and 5 both provide evidence of food limitation, because each figure demonstrates that given the opportunity Delta Smelt will consume more prey, and our previous work has linked decreased foraging success in Delta Smelt with decreased growth and condition factor [32]. Among the variables examined in our study, including salinity, temperature, turbidity, and mesozooplankton density, the latter was the variable with the strongest evidence for influencing foraging success in the wild. Thus, given both the direct evidence for food limitation in Delta Smelt (Figs 2 and 5, [32]), as well as the indirect evidence [28, 70, 71], it is important for the conservation of the species that our understanding of the decline of mesozooplankton in the SFE improves. Certainly, the loss of productive, shallow habitat during the 19^th^ and 20^th^ centuries is a likely contributor, but it largely predated the mesozooplankton decline in Fig 1 [30, 72]. In brackish water, the decline in mesozooplankton abundance after 1986 could be attributed to the invasion of the SFE by *P*. *amurensis*, both via competition for phytoplankton and predation [28, 42, 70, 73]. However, *P amurensis* does not occur below roughly 2 psu [74], so it cannot explain the mesozooplankton decline in freshwater (Fig 1). While *Corbicula fluminea*, another invasive bivalve, inhabits freshwater portions of the SFE, it was first introduced to the San Francisco area in 1946, so it coexisted with the relatively high mesozooplankton densities in the 1970s [75]. One possibility is that declining phosphorus resulting from more stringent water quality standards has slowed primary productivity in the SFE [76–78]. While the P limitation hypothesis is appealing because it could explain the productivity decline in both freshwater and brackish regions, including the substantial decline in brackish water before 1986, Jassby et al. [79] found that of 6000 measurements of soluble reactive P in the SFE, only 9 fell below concentrations that are considered limiting. Other possibilities are that high ammonia concentrations inhibit phytoplankton growth [80], limiting mesozooplankton from the bottom-up, that rapid increases in water exports in the late 1960s and early 1970s caused a decline in phytoplankton and mesozooplankton abundance [29, 81], or that chronic contamination has contributed to estuary-wide organism declines through sublethal pathways that are difficult to detect in the field [82]. Whatever the cause, if the decline in mesozooplankton can be reversed, overall foraging success of Delta Smelt will likely improve, particularly if productivity can be increased in freshwater during fall, winter and spring (Figs 2 and 5; [83]). This goal seems attainable, given that mesozooplankton densities were far higher in the 1970s, despite the presence of *C*. *fluminea* (Fig 1).

While our results indicate that Delta Smelt that migrate to brackish habitat have improved foraging success, we do not conclude that migratory fish will have higher fitness than freshwater residents. For both migratory and non-migratory life histories to be maintained evolutionarily, a fitness tradeoff must exist between the two strategies that depends on phenotype [1, 84]. For example, small body size is often associated with migratory life-history in fishes, as individuals exhibiting relatively poor growth adopt the relatively riskier life history strategy of migration [1, 14]. The risks, which include mortality due to predation and osmoregulatory challenges, can be offset by improvements in fitness (e.g., [16, 85]). However, for migration to increase fitness it must more than offset any decrease in survival rate, and it is unknown whether the improvement in foraging success described herein makes this true for Delta Smelt. Thus, we conclude that both migratory and freshwater resident life history strategies are likely important for population persistence, as individuals can pursue the life-history strategy that maximizes fitness for their phenotype, leading to more stable populations (i.e., the Sommer et al. contingent hypothesis [21, 86]).

Similarly, despite increased foraging success in brackish habitat, we do not conclude that increased salinity in the SFE would benefit Delta Smelt. On one hand, we detected no metabolic rate differences among Delta Smelt tested at salinities ranging from 0.4 to 12.0 psu, and Delta Smelt in brackish habitat outperformed the freshwater individuals in terms of foraging success. However, there appears to be an upper salinity limit to the benefits provided by brackish water of roughly 8 psu, and a foraging optimum appears to occur at 2–4 psu, although we note that salinity is conflated with turbidity (among other factors) in Fig 4. Delta Smelt seem to prefer salinities below 6 psu [87], so the foraging difficulties we observed at higher salinities provides a potential mechanism for this preference. Moreover, Feyrer et al. [88] demonstrated that Delta Smelt habitat and abundance decreases in drier, more saline years, and the current drought has coincided with an alarming decline in abundance (CDFW *unpublished data*). Thus, both because we cannot conclude that the migratory life-history strategy is superior, and because there is an upper limit to the salinity at which foraging benefits are realized, we do not suggest that increasing salinity would benefit Delta Smelt.

In summary, we observed significant improvements in foraging of Delta Smelt in brackish regions, consistent with other temperate anadromous and semi-anadromous fishes. However, there was a strong interaction with season, with greater foraging success in freshwater in summer, but far greater foraging success for the remainder of the year in brackish regions, due mainly to an increase in the number of prey items in Delta Smelt stomachs rather than their weight. This seasonal asynchronicity in foraging success likely benefits migratory Delta Smelt, allowing young fish to exploit relatively abundant prey in freshwater before moving to brackish habitat with more accessible prey. The fall decline in freshwater foraging success was likely due to a seasonal decline in mesozooplankton abundance, while the fall increase in foraging success in brackish habitat was due to an increase in foraging efficiency (i.e., a higher proportion of available mesozooplankton were eaten), but it is unclear why this occurred. We found little evidence that metabolic demand, either due to temperature or salinity, was driving the differences we observed in stomach fullness between the two habitat types. Thus, given the growing evidence of food limitation in Delta Smelt, an increase in pelagic productivity in the SFE would likely benefit the species.

## Supporting information

S1 Fig Mean prey mesozooplankton m^-3^ of Delta Smelt by year (1972–2015) in the SFE. Grey points represent freshwater (<0.55 psu mean salinity) and black points represent brackish habitat (≥0.55 psu mean salinity). Prey zooplankton includes all organisms in the mesozooplankton samples besides rotifers, crab zoea, and barnacle nauplii (i.e., only copepods and cladocera).
(TIF)

S2 Fig Weight of individual taxa in Delta Smelt stomachs as a percentage of total weight of stomach contents during Summer Townet. Grey bars represent stomach contents of Delta Smelt sampled in freshwater (<0.55; n = 143) and black bars represent stomach contents of Delta Smelt sampled in brackish habitat (≥0.55 psu; n = 105). Error bars are ±SE.
(TIF)

S3 Fig Weight of individual taxa in Delta Smelt stomachs as a percentage of total weight of stomach contents during Fall Midwater Trawl. Grey bars represent stomach contents of Delta Smelt sampled in freshwater (<0.55; n = 131) and black bars represent stomach contents of Delta Smelt sampled in brackish habitat (≥0.55 psu; n = 164). Error bars are ±SE.
(TIF)

S4 Fig Weight of individual taxa in Delta Smelt stomachs as a percentage of total weight of stomach contents during Spring Kodiak Trawl. Grey bars represent stomach contents of Delta Smelt sampled in freshwater (<0.55; grey bars; n = 402) and black bars represent stomach contents of Delta Smelt sampled in brackish habitat (≥0.55 psu; n = 347). Error bars are ±SE.
(TIF)

S5 Fig Mesozooplankton m^-3^ by month in the SFE. Triangular points are monthly averages from 1972–1986, circular points are from 1987–2015. Grey is freshwater (mean salinity <0.55), black is brackish water (≥0.55). Error bars are ±SE.
(TIF)

S1 File Standard metabolic rate: Fish maintenance, acclimation and measurements. (DOCX)

S2 File Supplemental results. (DOCX)

S3 File Stomach fullness data. (XLSX)

S4 File Freshwater functional response data. (XLSX)

S5 File Brackish functional response data. (XLSX)

S6 File Metabolic data. (XLSX)

S1 Table Delta Smelt collection. Sites where Delta Smelt were collected (n = 1,318), sample size (number of Delta Smelt collected), mean salinity (psu), mean temperature (°C), mean turbidity (NTU), and sampling site latitude and longitude. Standard deviations are in parentheses.
(DOCX)
