# Supplementary figures and images for: Foraging and metabolic consequences of semi-anadromy for an endangered estuarine fish
Source: PLoS One. 2017 Mar 14;12(3):e0173497. doi: 10.1371/journal.pone.0173497 (PMC5349674; doi:10.1371/journal.pone.0173497)

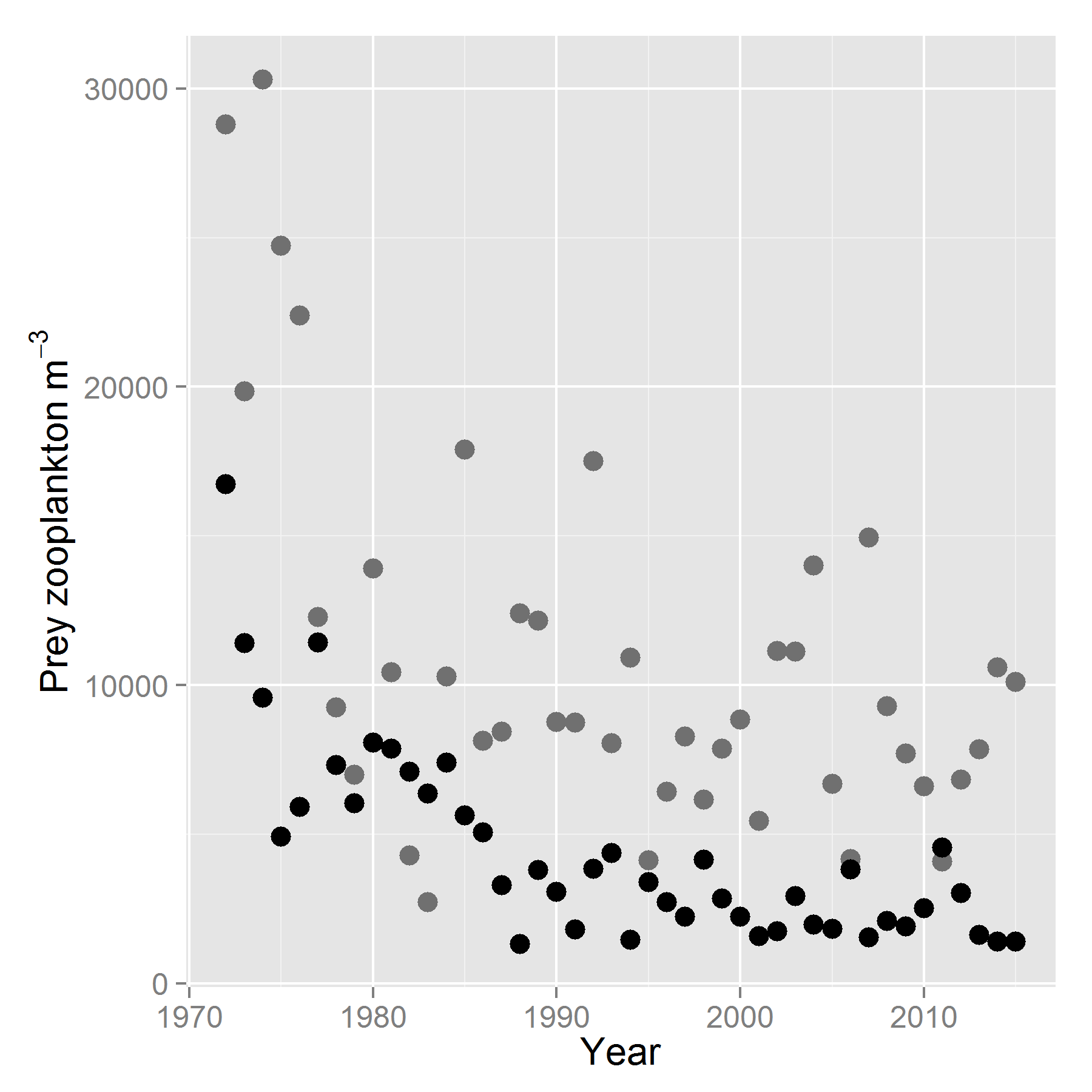

Supplement: S1 Fig — Grey points represent freshwater (<0.55 psu mean salinity) and black points represent brackish habitat (≥0.55 psu mean salinity). Prey zooplankton includes all organisms in the mesozooplankton samples besides rotifers, crab zoea, and barnacle nauplii (i.e., only copepods and cladocera). (TIF) [file pone.0173497.s001.tif]

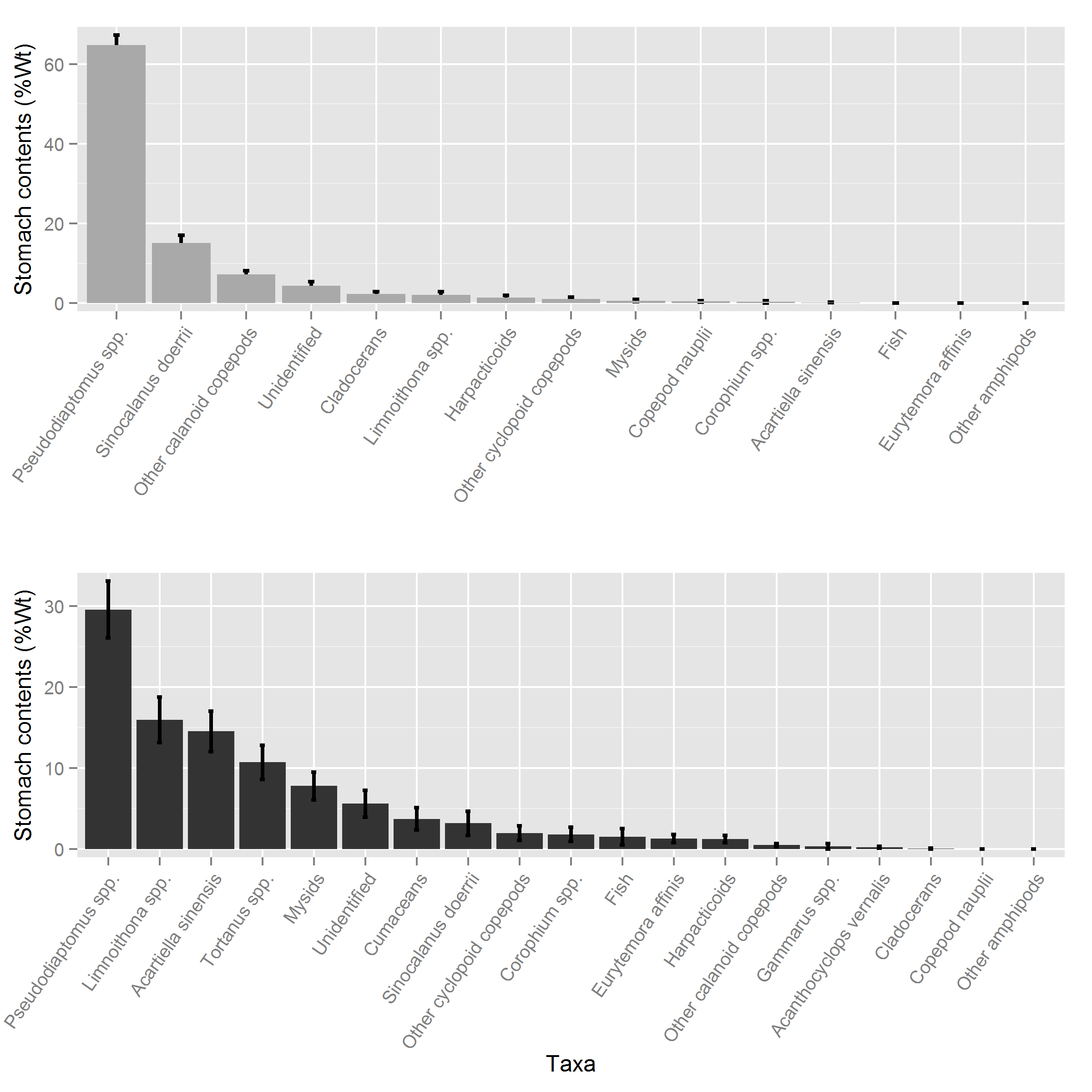

Supplement: S2 Fig — Grey bars represent stomach contents of Delta Smelt sampled in freshwater (<0.55; n = 143) and black bars represent stomach contents of Delta Smelt sampled in brackish habitat (≥0.55 psu; n = 105). Error bars are ±SE. (TIF) [file pone.0173497.s002.tif]

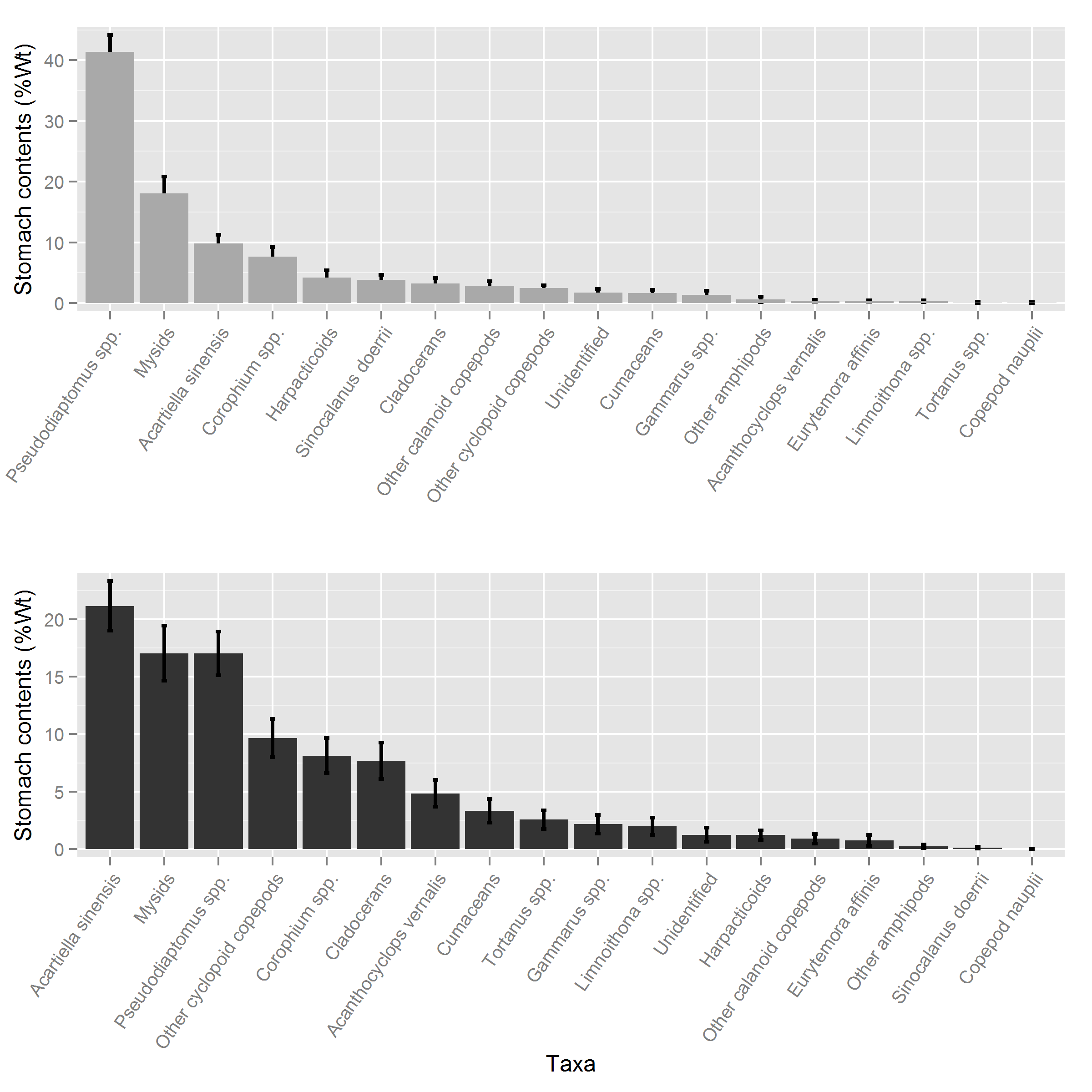

Supplement: S3 Fig — Grey bars represent stomach contents of Delta Smelt sampled in freshwater (<0.55; n = 131) and black bars represent stomach contents of Delta Smelt sampled in brackish habitat (≥0.55 psu; n = 164). Error bars are ±SE. (TIF) [file pone.0173497.s003.tif]

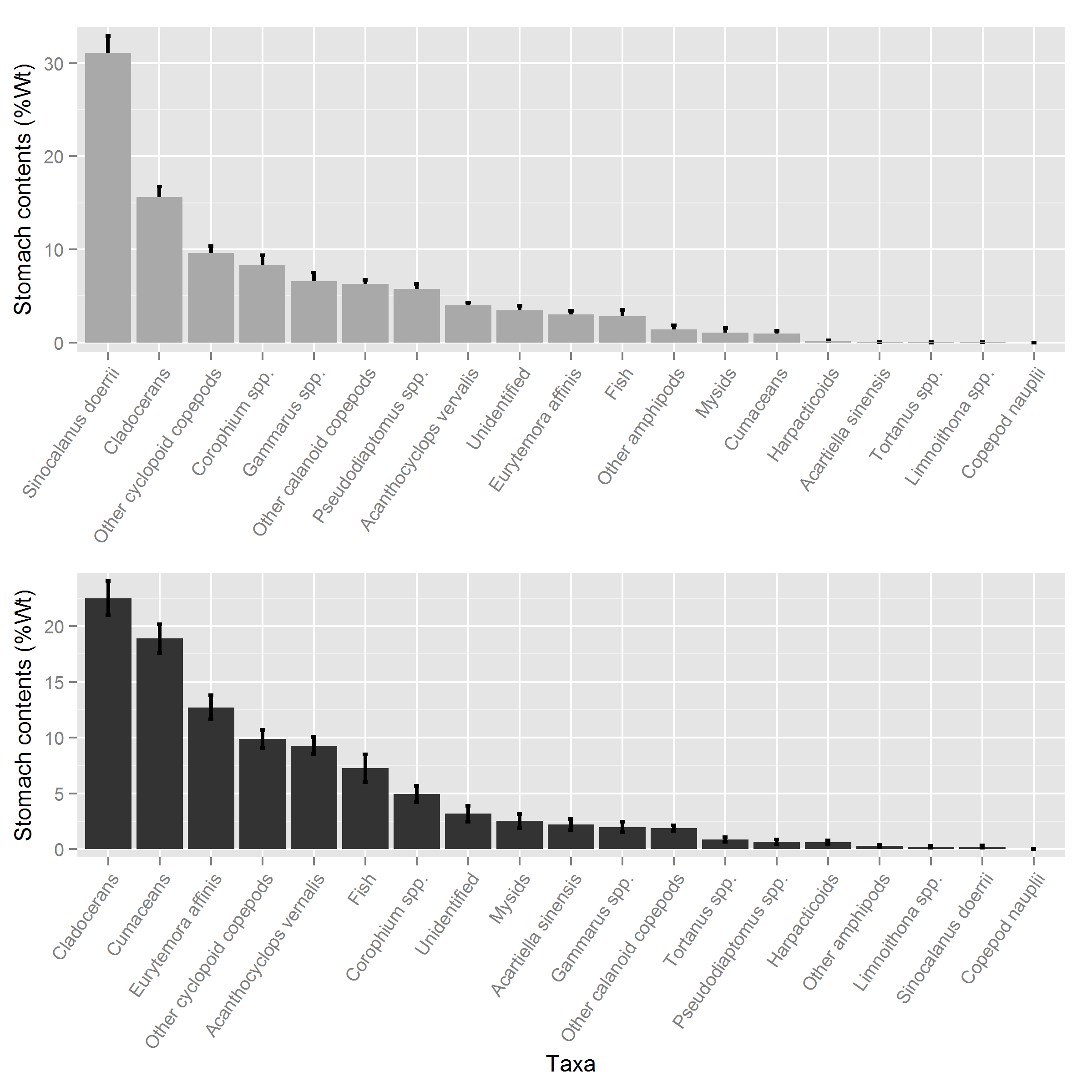

Supplement: S4 Fig — Grey bars represent stomach contents of Delta Smelt sampled in freshwater (<0.55; grey bars; n = 402) and black bars represent stomach contents of Delta Smelt sampled in brackish habitat (≥0.55 psu; n = 347). Error bars are ±SE. (TIF) [file pone.0173497.s004.tif]

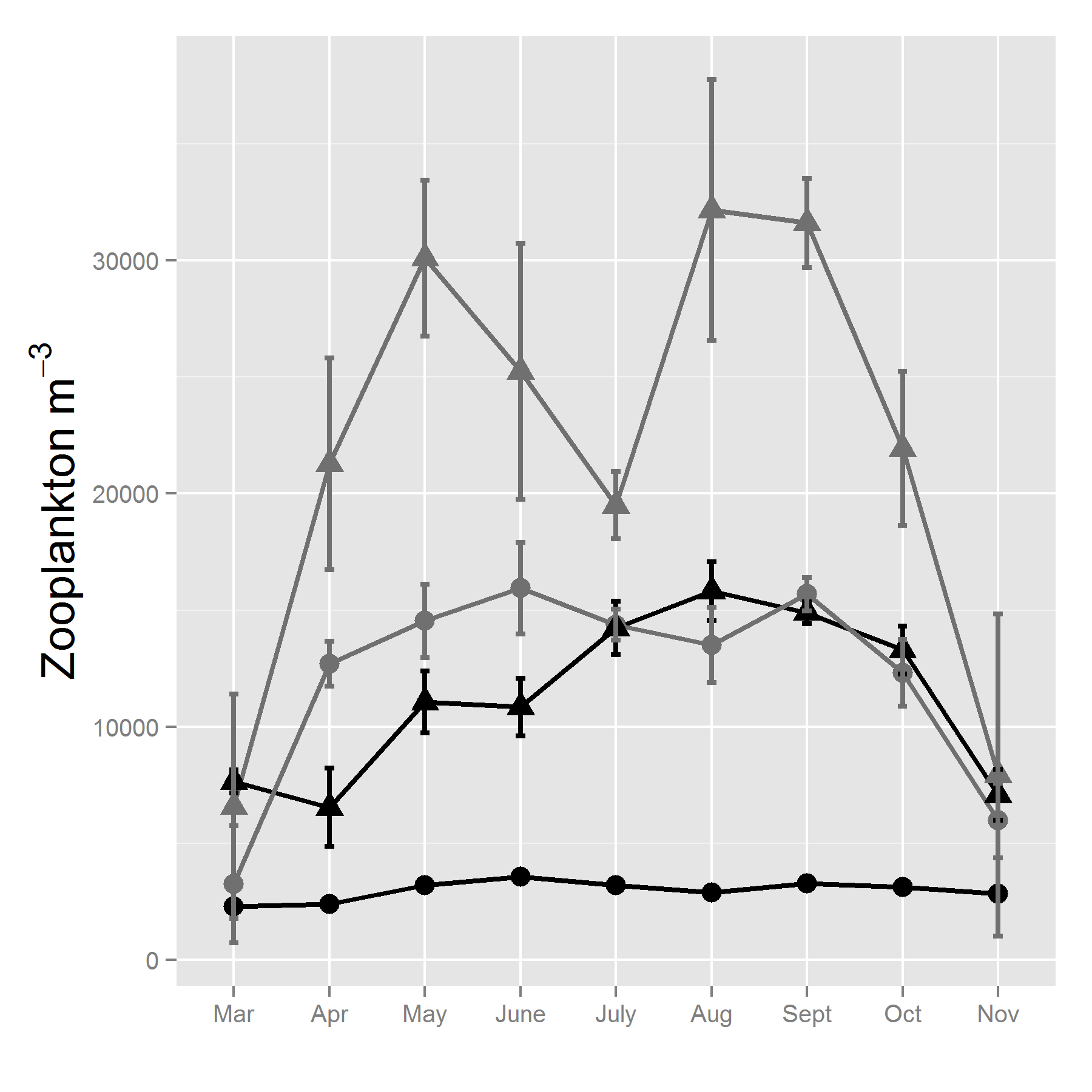

Supplement: S5 Fig — Triangular points are monthly averages from 1972–1986, circular points are from 1987–2015. Grey is freshwater (mean salinity <0.55), black is brackish water (≥0.55). Error bars are ±SE. (TIF) [file pone.0173497.s005.tif]
