# Supplementary material for: Foraging and metabolic consequences of semi-anadromy for an endangered estuarine fish
Source: PLoS One. 2017 Mar 14;12(3):e0173497. doi: 10.1371/journal.pone.0173497 (PMC5349674; doi:10.1371/journal.pone.0173497)
Supplement: S1 File — (DOCX) [file pone.0173497.s006.docx]

Standard metabolic rate: Fish maintenance and acclimation

On Aug 20, 2015 375 Delta Smelt (200 days-post-hatch) were transported from UC Davis’ Fish Conservation & Culture Laboratory (FCCL) near Byron, CA to UC Davis’ Fish Ecophysiology lab, Davis, CA in three 76 L insulated carboys (125 fish in each). Before transportation, pure oxygen was bubbled into each carboy to saturation, thus maintaining dissolved oxygen above 85% throughout the ~2 h trip. In addition, salinity was raised from 0.4 psu (at which the fish were cultured) to 3 psu to reduce handling stress, and a prophylactic stress-coat treatment Novaqua Plus^TM^ was applied to protect the slime coat of the fish (Swanson et al. 1996). Fish were gently poured from each carboy into three indoor, black, circular 300 L tanks. Each tank was filled with 190 L well water at 0.4 psu and loosely covered with a mesh lid covered in opaque black plastic (photoperiod = 11L:13D, daytime light level 1 lux). A combination of flow-through (~10 L/min) and wet/dry biological filtration was used to maintain water quality for the first five weeks. On Sept 29 a salinity treatment was randomly assigned to each tank (0.4, 2.0 or 12.0 psu), the 2.0 and 12.0 tanks were transitioned to wet/dry biological filtration only (i.e., recirculation), and treatment salinities were gradually reached over the following week. Salinity was increased by daily replacing water in the 2.0 and 12.0 psu treatment tank sumps with a concentrated solution of Instant Ocean Salt dissolved in well water (~50 psu). Ammonia, nitrate, and nitrite were monitored daily for five weeks after switching the systems to recirculation, and intermittently thereafter using Aquarium Pharmaceuticals Inc. kits. We performed ~60% water changes a minimum of twice per week for all treatments. We used automatic feeders to feed the fish ad libitum throughout each day with a mixture of two parts O.range Grow-S fish hatchery diet (INVE Aquaculture) and one part Hikari plankton (Kyorin Food Industries LTD). Salinity, temperature and specific conductivity were monitored daily using a YSI 85 meter (Main text Table 2).

Standard metabolic rate: Measurements

We measured standard metabolism from Nov 3-19, 2015, following a minimum 21 day acclimation for all three treatment salinities by the start of the experiment. The respirometers, made of acrylic plastic, measured 14.8 × 7.7 × 9.7 cm (1.1 L). A black, rubber gasket was sandwiched between the lid and the chamber, and a seal was made by applying vacuum grease to the gasket and bolting the lid down at six points. To begin the measurements, four fish were scooped individually from the tank using an opaque plastic cup and poured into each of the four respirometers (~a 10 s process per fish), and the chambers were sealed. Following introductions, the plastic sheeting was closed to darken the water bath (0 lux). Fish in the holding tanks were fed after the introductions to ensure that the fish in the respirometers were in a postabsorptive state (Cech 1990). Fish were given 30 min to acclimate to the chambers, during which water was gently pumped through the chambers using the ‘flush’ cycle described below.

We used intermittent respirometry to quantify standard metabolic rate, which had three cycles: measurement, flush, and wait (Chabot et al. 2016). Chabot et al. (2016) recommends an experimental duration of 24 h for the measurement of standard metabolic rate. However, in previous experiments Delta Smelt died during the night in the respirometers (>50% mortality, D.E. Cocherell, *unpublished data*), so we used a measurement duration of 5 h. The flush and measurement/wait cycles alternated between two independent circulation systems, one that was open to the water bath (flush) and one that was internal (measurement/wait), both of which moved water gently past the fish at all times (~1-2 cm/s). Rainbird© 075 solenoid valves were used to switch between the flush and wait/measurement cycles, which was automated using Loligo Systems AutoResp software. Following the initial 30 min acclimation period (flush), the cycles began with a 37 min measurement cycle, during which the chamber was sealed from the water bath and oxygen loss due to respiration was quantified with a galvanic oxygen probe (MINI-DO Loligo Systems). During this period water was recirculated within each chamber with submersible brushless DC pumps, and dissolved oxygen measurements were recorded every second. A 10 min flush cycle followed, during which the chamber was unsealed and water was pumped from the water bath through the chamber, returning dissolved oxygen to saturation. The chamber was then sealed to begin the 3 min wait cycle, during which oxygen depletion became linear. The cycle was repeated five times for each fish before measurements ceased and the wet weight of each fish was obtained. The mean r^2^ for lines fit to the oxygen~time relationship for each measurement cycle was 0.999, and the minimum r^2^ was 0.987, indicating highly linear relationships. The duration of cycle periods was based on previous studies in a similar chamber.

The data from the trials both with and without fish were inputted into the following equation to calculate oxygen demand:

*O* = [(*O_2_* (initial) – *O_2_* (final) * *V*] / *T*

where *O* is oxygen demand (mg O_2_ h^–1^), *O_2_* (initial) is the concentration of oxygen at the start of the interval inside the chamber (mg O_2_ L^–1^), *O_2_* (final) is the concentration of oxygen at the end of the interval inside the chamber (mg O_2_ L^–1^), *V* is the chamber’s volume, *T* is the elapsed time during the measurement (h). To calculate metabolic demand for each fish, we subtracted *O* without fish (blank) from *O* with fish.
