# Supplementary material for: Foraging and metabolic consequences of semi-anadromy for an endangered estuarine fish
Source: PLoS One. 2017 Mar 14;12(3):e0173497. doi: 10.1371/journal.pone.0173497 (PMC5349674; doi:10.1371/journal.pone.0173497)
Supplement: S2 File — (DOCX) [file pone.0173497.s007.docx]

Using only the final four measurement cycles, weight influenced standard metabolic rate (ANCOVA, F_1, 58_ = 17.2236, P = 0.0001), and salinity did not (ANCOVA, F_2, 58_ = 1.1515, P = 0.3233). Treatment means, adjusted by mean weight of each treatment, were 0.467, 0.537, and 0.467 mg O_2_ h^-1^ for the 0.4, 2.0 and 12.0 psu treatments, respectively. Unadjusted for weight, treatment means were 0.467, 0.537, and 0.467 mg O_2_ h^-1^. Similarly, using only the final two measurement cycles, weight influenced standard metabolic rate (ANCOVA, F_1, 58_ = 11.4630, P = 0.0013) and salinity did not (ANCOVA, F_1, 58_ = 0.3249). Treatment means, adjusted by mean weight of each treatment, were 0.420, 0.514, and 0.450 mg O_2_ h^-1^ for the 0.4, 2.0 and 12.0 psu treatments, respectively. Unadjusted for weight, treatment means were 0.433, 0.510, and 0.443 mg O_2_ h^-1^. Using all five measurement cycles, mean standard metabolic rate values (unadjusted for differences in treatment weight) were 0.502, 0.551, and 0.470 mg O_2_ h^-1^.
