# Supplementary material for: Foraging and metabolic consequences of semi-anadromy for an endangered estuarine fish
Source: PLoS One. 2017 Mar 14;12(3):e0173497. doi: 10.1371/journal.pone.0173497 (PMC5349674; doi:10.1371/journal.pone.0173497)
Supplement: S1 Table — Sites where Delta Smelt were collected (n = 1,318), sample size (number of Delta Smelt collected), mean salinity (psu), mean temperature (°C), mean turbidity (NTU), and sampling site latitude and longitude. Standard deviations are in parentheses. (DOCX) [file pone.0173497.s012.docx]

| Station | n | Salinity (psu) | Temperature (°C) | Turbidity (NTU) | Latitude | Longitude |
| --- | --- | --- | --- | --- | --- | --- |
| 340 | 3 | 14.9 (1.3) | 14.3 (5.1) | 42.7 (17.6) | 38 6 20.0 | -122 16 10.0 |
| 405 | 2 | 3.6 (3.3) | 16.0 (4.4) | 134.4 (43.0) | 38 1 59.9 | -122 9 21.6 |
| 407 | 1 | 8.9 (na) | 14.0 (na) | 14.2 (na) | 38 1 59.9 | -122 8 31.2 |
| 411 | 10 | 2.8 (2.8) | 14.2 (3.1) | 75.8 (38.6) | 38 3 29.9 | -122 4 8.4 |
| 412 | 2 | 7.7 (0.0) | 14.5 (0.0) | 13.9 (0.0) | 38 3 50.0 | -122 3 21.6 |
| 413 | 3 | 4.0 (0.0) | 20.0 (0.0) | 77.0 (0.0) | 38 3 50.1 | -122 2 31.2 |
| 416 | 1 | 10.8 (na) | 18.6 (na) | 8.6 (na) | 38 4 5.9 | -122 5 34.8 |
| 417 | 1 | 10.6 (na) | 10.3 (na) | 12.5 (na) | 38 4 50.9 | -122 4 37.2 |
| 418 | 5 | 7.5 (4.1) | 19.1 (3.6) | 72.2 (71.8) | 38 5 35.2 | -122 4 15.6 |
| 501 | 17 | 7.2 (3.0) | 13.1 (5.8) | 22.2 (11.6) | 38 3 40.0 | -122 1 30.0 |
| 502 | 3 | 5.0 (2.5) | 16.9 (5.5) | 37.2 (23.0) | 38 3 29.9 | -122 0 10.8 |
| 503 | 3 | 2.3 (0.0) | 20.7 (0.0) | 56.0 (0.0) | 38 3 29.9 | -121 59 9.6 |
| 504 | 8 | 4.7 (4.4) | 12.7 (4.0) | 27.2 (15.5) | 38 3 29.9 | -121 58 19.2 |
| 505 | 2 | 1.4 (0.6) | 16.3 (3.7) | 30.5 (6.4) | 38 3 20.2 | -121 57 21.6 |
| 507 | 30 | 0.5 (1.0) | 18.5 (2.4) | 36.1 (7.6) | 38 2 60.0 | -121 56 9.6 |
| 508 | 20 | 3.8 (2.7) | 13.6 (5.2) | 16.8 (4.8) | 38 2 49.9 | -121 55 22.8 |
| 509 | 4 | 1.0 (1.6) | 16.2 (4.0) | 15.3 (1.6) | 38 2 39.8 | -121 54 36.0 |
| 510 | 2 | 1.7 (2.3) | 20.0 (3.0) | 13.7 (4.0) | 38 2 49.9 | -121 53 31.2 |
| 511 | 2 | 1.4 (1.9) | 17.9 (0.5) | 13.3 (5.9) | 38 2 60.0 | -121 52 58.8 |
| 512 | 6 | 2.4 (1.1) | 18.7 (1.1) | 24.9 (13.7) | 38 3 20.2 | -121 52 30.0 |
| 513 | 46 | 2.3 (2.4) | 14.3 (4.2) | 26.6 (14.5) | 38 3 40.0 | -121 51 50.4 |
| 515 | 7 | 6.8 (4.1) | 15.9 (5.4) | 47.0 (15.1) | 38 5 28.0 | -122 0 50.4 |
| 516 | 2 | 4.0 (2.6) | 14.6 (7.1) | 66.4 (63.1) | 38 4 35.0 | -121 59 31.2 |
| 517 | 9 | 4.5 (3.0) | 12.1 (3.2) | 27.9 (17.2) | 38 4 9.8 | -121 58 40.8 |
| 518 | 18 | 2.7 (1.7) | 15.8 (4.6) | 36.6 (27.0) | 38 4 8.0 | -121 57 21.6 |
| 519 | 98 | 4.9 (3.3) | 14.3 (4.9) | 41.4 (22.9) | 38 4 25.0 | -121 55 58.8 |
| 520 | 23 | 2.1 (1.2) | 11.3 (4.3) | 14.4 (5.6) | 38 01 58.1 | -121 52 09.5 |
| 601 | 1 | 8.1 (na) | 14.4 (na) | 13.5 (na) | 38 6 11.9 | -122 1 58.8 |
| 602 | 40 | 4.2 (3.0) | 17.6 (4.0) | 85.8 (55.3) | 38 6 58.0 | -122 1 51.6 |
| 603 | 1 | 11.9 (na) | 10.3 (na) | 26.3 (na) | 38 6 47.9 | -122 2 31.2 |
| 605 | 2 | 7.3 (0.3) | 10.4 (0.3) | 23.9 (12.2) | 38 8 49.9 | -122 3 21.6 |
| 606 | 111 | 4.3 (2.2) | 11.3 (3.4) | 52.5 (33.1) | 38 10 9.8 | -122 1 19.2 |
| 609 | 121 | 2.6 (1.4) | 13.4 (4.2) | 50.2 (23.4) | 38 10 01.9 | -121 56 16.8 |
| 610 | 18 | 1.9 (1.4) | 14.2 (4.4) | 45.7 (11.8) | 38 07 07.7 | -121 53 21.1 |
| 701 | 1 | 2.3 (na) | 13.9 (na) | 12.5 (na) | 38 3 50.0 | -121 49 40.8 |
| 703 | 27 | 1.8 (0.7) | 13.2 (3.7) | 25.9 (5.1) | 38 3 40.0 | -121 47 49.2 |
| 704 | 70 | 0.6 (0.6) | 13.7 (4.3) | 19.1 (13.1) | 38 4 9.9 | -121 46 30.0 |
| 705 | 26 | 0.4 (0.4) | 10.3 (2.5) | 12.8 (3.1) | 38 4 50.2 | -121 45 21.6 |
| 706 | 77 | 0.3 (0.4) | 10.2 (2.3) | 17.0 (11.7) | 38 5 30.1 | -121 44 20.4 |
| 707 | 24 | 0.1 (0.1) | 11.3 (2.8) | 26.4 (10.3) | 38 6 50.1 | -121 42 21.6 |
| 711 | 3 | 0.1 (0.0) | 12.9 (0.5) | 38.1 (6.5) | 38 10 30.0 | -121 40 8.4 |
| 712 | 3 | 0.1 (0.0) | 12.1 (0.0) | 23.8 (0.0) | 38 11 36.5 | -121 37 21.4 |
| 713 | 22 | 0.1 (0.0) | 11.0 (2.0) | 26.4 (10.7) | 38 12 20.2 | -121 39 28.8 |
| 715 | 42 | 0.1 (0.0) | 12.1 (3.4) | 28.7 (11.3) | 38 13 19.9 | -121 40 19.2 |
| 716 | 77 | 0.1 (0.0) | 16.8 (4.4) | 31.9 (9.5) | 38 14 28.8 | -121 41 8.4 |
| 719 | 194 | 0.2 (0.0) | 13.1 (5.1) | 34.9 (12.9) | 38 20 00.2 | -121 38 51.1 |
| 721 | 30 | 0.1 (0.0) | 19.8 (1.2) | 40.4 (12.6) | 38 16 06.4 | -121 42 11.1 |
| 723 | 1 | 0.1 (na) | 21.1 (na) | 23.4 (na) | 38 14 14.0 | -121 40 23.0 |
| 796 | 1 | 0.4 (na) | 13.4 (na) | 44.3 (na) | 38 28 24.8 | -121 35 04.0 |
| 797 | 59 | 0.3 (0.0) | 19.9 (4.5) | 42.6 (16.1) | 38 24 17.0 | -121 36 56.2 |
| 801 | 22 | 3.7 (1.0) | 11.3 (3.8) | 12.5 (5.0) | 38 3 20.2 | -121 50 49.2 |
| 802 | 1 | 0.2 (na) | 21.2 (na) | 49.9 (na) | 38 2 10.0 | -121 50 9.6 |
| 804 | 13 | 1.2 (1.0) | 13.1 (3.8) | 17.1 (5.4) | 38 1 19.9 | -121 47 38.4 |
| 806 | 1 | 0.6 (na) | 8.5 (na) | 10.4 (na) | 38 1 40.1 | -121 45 21.6 |
| 807 | 2 | 0.7 (0.0) | 8.9 (0.0) | 8.1 (0.0) | 38 1 50.2 | -121 43 51.6 |
